# Targeting interferon response genes sensitizes aromatase inhibitor resistant breast cancer cells to estrogen-induced cell death

**DOI:** 10.1186/s13058-014-0506-7

**Published:** 2015-01-15

**Authors:** Hye Joung Choi, Asona Lui, Joshua Ogony, Rifat Jan, Peter J Sims, Joan Lewis-Wambi

**Affiliations:** Department of Cancer Biology, University of Kansas Medical Center, Kansas City, 66160 KS USA; Department of Physiology, University of Kansas Medical Center, Kansas City, 66160 KS USA; Cancer Biology Program, Research Institute of Fox Chase Cancer Center, Philadelphia, 19111 PA USA; Department of Pathology and Laboratory Medicine, University of Rochester, Rochester, 14642 NY USA

## Abstract

**Introduction:**

Estrogen deprivation using aromatase inhibitors (AIs) is currently the standard of care for postmenopausal women with hormone receptor-positive breast cancer. Unfortunately, the majority of patients treated with AIs eventually develop resistance, inevitably resulting in patient relapse and, ultimately, death. The mechanism by which resistance occurs is still not completely known, however, recent studies suggest that impaired/defective interferon signaling might play a role. In the present study, we assessed the functional role of IFITM1 and PLSCR1; two well-known interferon response genes in AI resistance.

**Methods:**

Real-time PCR and Western blot analyses were used to assess mRNA and protein levels of IFITM1, PLSCR1, STAT1, STAT2, and IRF-7 in AI-resistant MCF-7:5C breast cancer cells and AI-sensitive MCF-7 and T47D cells. Immunohistochemistry (IHC) staining was performed on tissue microarrays consisting of normal breast tissues, primary breast tumors, and AI-resistant recurrence tumors. Enzyme-linked immunosorbent assay was used to quantitate intracellular IFNα level. Neutralizing antibody was used to block type 1 interferon receptor IFNAR1 signaling. Small interference RNA (siRNA) was used to knockdown IFITM1, PLSCR1, STAT1, STAT2, IRF-7, and IFNα expression.

**Results:**

We found that IFITM1 and PLSCR1 were constitutively overexpressed in AI-resistant MCF-7:5C breast cancer cells and AI-resistant tumors and that siRNA knockdown of IFITM1 significantly inhibited the ability of the resistant cells to proliferate, migrate, and invade. Interestingly, suppression of IFITM1 significantly enhanced estradiol-induced cell death in AI-resistant MCF-7:5C cells and markedly increased expression of p21, Bax, and Noxa in these cells. Significantly elevated level of IFNα was detected in AI-resistant MCF-7:5C cells compared to parental MCF-7 cells and suppression of IFNα dramatically reduced IFITM1, PLSCR1, p-STAT1, and p-STAT2 expression in the resistant cells. Lastly, neutralizing antibody against IFNAR1/2 and knockdown of STAT1/STAT2 completely suppressed IFITM1, PLSCR1, p-STAT1, and p-STAT2 expression in the resistant cells, thus confirming the involvement of the canonical IFNα signaling pathway in driving the overexpression of IFITM1 and other interferon-stimulated genes (ISGs) in the resistant cells.

**Conclusion:**

Overall, these results demonstrate that constitutive overexpression of ISGs enhances the progression of AI-resistant breast cancer and that suppression of IFITM1 and other ISGs sensitizes AI-resistant cells to estrogen-induced cell death.

**Electronic supplementary material:**

The online version of this article (doi:10.1186/s13058-014-0506-7) contains supplementary material, which is available to authorized users.

## Introduction

Aromatase inhibitors (AIs) are more effective than the antiestrogen tamoxifen at inhibiting the growth and proliferation of estrogen receptor (ER)-positive breast cancer [1] and these agents are now front-line treatments for postmenopausal women with hormone receptor-positive breast cancer in both the adjuvant and metastatic setting [2,3]. AIs suppress estrogen synthesis in postmenopausal women by inhibiting the aromatase enzyme, which catalyzes the conversion of androgens to estrogens [1,2,4,5]. Unfortunately, the majority of patients treated with AIs eventually develop resistance to these drugs [6] and when resistance occurs it is unclear which endocrine therapy is the most appropriate. Recently, there has been increasing clinical evidence to suggest that 17β-estradiol (E_2_) would be an appropriate and effective treatment option for postmenopausal patients with AI-resistant breast cancer [7,8]. Indeed, preclinical studies from our laboratory [9-12] and other investigators [13,14]) have previously shown that long term estrogen deprivation of ER-positive MCF-7 breast cancer cells causes them to lose their dependency on estradiol for proliferation, which recapitulates acquired resistance to aromatase inhibitors in postmenopausal women, and that these AI-resistant breast cancer cells paradoxically undergo apoptosis in the presence of estradiol [10-12,15,16]. The ability of estradiol to induce apoptosis in AI-resistant breast cancer cells was previously shown to be mediated, in part, by the mitochondria death pathway [11]; however, more recent findings suggest that dysregulation of the interferon signaling pathway might also play a role in estradiol-induced cell death [17].

Interferons (IFNs) are a class of glycoproteins known as cytokines that are produced by immune cells of most vertebrates and are secreted in response to viral infections, tumors, and other pathogenic microbial agents [18]. IFNs diffuse to the surrounding cells and bind to high affinity cell surface type I (IFNα/β) and type II (IFNγ) receptors (IFNAR1/2), leading to phosphorylation and activation of JAK1, JAK2 and Tyk2. Activated JAKs phosphorylate and activate STAT1 and STAT2, resulting in the formation of STAT1-STAT1 homodimers and STAT1-STAT2 heterodimers. The dimers are transported to the nucleus by importins and bind to IFN-stimulated response elements (ISREs) to activate the transcription of interferon-stimulated genes (ISGs), such as *IFITM1, PLSCR1, STAT1, IFI27* and *IFIT1* [18-20]. The interferon signaling pathway plays an important role in the proper functioning of the immune system [21] and there is strong evidence that its dysregulation, resulting in constitutive overexpression of ISGs contributes to tumorigenesis [22] and possibly drug resistance [23]. Indeed, our laboratory has previously shown through microarray analysis that immune response and interferon signaling pathways are significantly altered in AI-resistant breast cancer cells and that several interferon response genes including *IFITM1, PLSCR1* and *STAT1* are constitutively overexpressed in AI-resistant breast cancer cells [17,24]. At present, however, the functional significance of the interferon signaling pathway in AI-resistance or its potential involvement in estradiol-induced cell death is not known.

Interferon-inducible transmembrane protein 1 (IFITM1) is a cell surface 17 kDa membrane protein that is a member of the IFN-inducible transmembrane protein family that includes IFITM2, IFITM3 and IFITM5 [25,26]. The IFITM1 gene is located on the short arm of chromosome 11 (11p15.5) and is 3,956 bases in length. IFITM1 expression is highly induced by IFNα and IFNβ to a lesser extent, IFNγ [27]. IFITM1 was initially identified as Leu13, a leukocyte antigen that is part of a membrane complex involved in the transduction of antiproliferative and homotypic adhesion signals in lymphocytes [28,29]. IFITM1 is also known to play a critical role in blocking early stages of viral replication and it potently restricts entry and infections by a number of highly pathogenic viruses, including HIV-1, filovirus, and SARS coronavirus [30]. More recently, there has been increasing evidence to suggest that high expression of IFITM1 plays a role in the progression of several cancers including head and neck cancer, serous ovarian cancer, gastric cancer and colorectal cancer [29,31-33]. However, its role in breast cancer or endocrine resistance is not known.

Phospholipid scramblase 1 (PLSCR1) is a 35 kDa multiply palmitoylated protein that is localized in the cell membrane and is responsible for mediating the translocation of negatively-charged phospholipids from the inner-leaflet of the plasma membrane to the outer-leaflet during cellular injury and apoptosis [34,35]. PLSCR1 is highly induced by type 1 IFNs and it plays an antagonistic role in leukemia [36] and ovarian cancer [37]; however, its role in breast cancer is unknown.

In the present study, we investigated the functional role of IFITM1 and PLSCR1 in AI-resistant breast cancer. We found that IFITM1 and PLSCR1 were constitutively overexpressed in AI-resistant MCF-7:5C breast cancer cells and AI-resistant tumors and that knockdown of IFITM1 significantly reduced their ability to proliferate, invade, and migrate. Most interestingly, we found that suppression of IFITM1 sensitized AI-resistant MCF-7:5C cells to estradiol-induced cell death. Suppression of IFNα level via siRNA knockdown of IRF-7 confirmed that the constitutive overexpression of IFITM1 and PLSCR1 in the resistant cells was driven by increased intracellular levels of IFNα. Further analysis using neutralizing antibody against IFNAR and siRNA knockdown of STAT1 and STAT2 revealed complete suppression of IFITM1, PLSCR1, p-STAT1 and p-STAT2 in the resistant cells, thus confirming a critical role for canonical IFNα signaling in the regulation of IFITM1 and other ISGs in the resistant cells.

## Methods

### Reagents

RPMI 1640 and fetal bovine serum (FBS) were obtained from Invitrogen Inc. (Grand Island, NY, USA). The antibiotic/antimycotic solution (containing 10,000 U/mL penicillin and 10 mg/mL streptomycin, 25 μg/mL of Fungizone®), NEAA (MEM Non-Essential Amino Acids), L-glutamine, and TrypLE (containing trypsin and ethylenediaminetetraacetic acid (EDTA)) were obtained from Invitrogen. Insulin (bovine pancrease), human recombinant interferon-alpha (IFNα) and 17β-estradiol (E_2_) were obtained from Sigma-Aldrich Co. (St. Louis, MO, USA). E_2_ was dissolved in ethanol at a stock concentration of 1 μM (10^−6^ M) and stored at −20°C. Anti-PLSCR1, anti-IFITM1, anti-IRF-7, anti-STAT1, anti-Bax, anti-Noxa, anti-PUMA, anti-p53, anti-p21, anti-Lamin B, anti-IFNAR (α − IFNAR), anti-p-STAT2 (Tyr690) and anti-ERα antibodies were purchased from Santa Cruz Technology Inc. (Santa Cruz, CA, USA); anti- poly ADP ribose polymerase (PARP) and anti-p-STAT1 (Y701) were purchased from Cell Signaling Technology (Beverly, MA, USA), and anti-β-actin was purchased from Sigma-Aldrich. PLSCR1 monoclonal antibody 4D2 was a kind gift from Dr. Peter Sims (University of Rochester, NY, USA).

### Cell lines and culture conditions

The ER-positive hormone-dependent human breast cancer cell lines, MCF-7 and T47D, were originally obtained from the American Type Culture Collection (Manassas, VA, USA) and were maintained in full serum medium composed of RPMI-1640 medium, 10% FBS, 2 mM glutamine, penicillin at 100 U/mL, streptomycin at 100 μg/mL, 1× NEAA (Invitrogen) and bovine insulin at 6 ng/mL (Sigma-Aldrich). The long term estrogen deprived human breast cancer cell line MCF-7:5C [9,12] was cloned from parental MCF-7 cells following long term (>12 months) culture in estrogen-free medium composed of phenol red-free RPMI-1640, 10% FBS treated three times with dextran-coated charcoal (SFS), 2 mM glutamine, bovine insulin at 6 ng/mL, penicillin at 100 U/mL, streptomycin at 100 μg/mL, and 1× NEAA. The MCF-7:5C cell line was used as a model of AI resistance because it proliferates despite being deprived of estrogen [9,12]. Cells were cultured at 37°C under 5% CO_2_ and were sub-cultured every three to four days.

### MTT assay

For determining cell viability, the 3-(4,5-dimethylthiazol-2-Yl)-2,5-diphenyltetrazolium bromide (MTT) assay was used. MCF-7 and MCF-7:5C cells were seeded onto 24-well plates at a density of 5 × 10^4^ cells per well in culture media and incubated until about 60% to 70% confluency, before the start of experimental treatments. The stock solution of E_2_ was diluted in the culture medium before addition to each well at desired final concentrations, and the treatments usually lasted 24 hours. Following the treatments as indicated, 50 μl of MTT solution (at 5 mg/mL) was added to each well at a final concentration of 500 μg/mL, and the mixture was further incubated for four hours at 37°C. An aliquot (500 μl) of the solubilizing solution (dimethyl sulfoxide (DMSO):ethanol, 1:1, v:v) was then added to each well, and the absorbance was read with a UV max microplate reader (Molecular Device, Palo Alto, CA, USA) at 560 nm. The relative cell density was expressed as a percentage of the control that was not treated with E_2_.

### Western blotting

For Western blotting, cells were washed first and then suspended in 100 μL lysis buffer (RIPA buffer, 150 mM NaCl, 1.0% IGEPAL® CA-630, 0.5% sodium deoxycholate, 0.1% SDS, 50 mM Tris, pH 8.0, protease inhibitor cocktail, and phosphatase inhibitor). The amount of proteins was determined using the Bio-Rad protein assay (Bio-Rad, Hercules, CA, USA). An equal amount of proteins was loaded in each lane. The proteins were separated by 4% to 12% SDS–polyacrylamide gel electrophoresis (SDS–PAGE) and electrically transferred to a polyvinylidene difluoride membrane (Bio-Rad). After blocking the membrane using 5% non-fat milk, target proteins were detected using specific antibodies. Thereafter, horseradish peroxidase (HRP)-conjugated anti-rabbit (or anti-mouse) immunoglobulin G (IgG) was applied as the secondary antibody and the positive bands were detected using Amersham ECL Plus Western blotting detection reagents (GE Health Care, Piscataway, NJ, USA).

### Nuclear and cytoplasmic fractionation

For protein localization, the nuclear and cytosolic fractions were prepared using the cytosolic/nuclear fractionation kit obtained from Biovision Inc. (Mountain View, CA, USA), by following the instructions of the manufacturer. Briefly, cells were suspended in hypotonic buffer and lysed with the proprietary detergent from the kit. Samples were spun at 800 × *g* for 10 minutes at 4°C. The supernatant was collected, spun five minutes at 16,000 × *g* to remove any remaining nuclei, and then transferred to a new microtube (cytosolic protein fraction). The original pellet was resuspended in the nuclear extraction buffer and then incubated on ice for 40 minutes with occasional vortexing. After salt extraction, the nuclear pellet was centrifuged at 16,000 × *g* for 10 minutes, and the supernatant was saved as the nuclear extract. Extracts were stored in aliquots in −80°C until use.

### Annexin V staining for apoptosis

An annexin V–fluorescein isothiocyanate (FITC)-labeled Apoptosis Detection Kit I (Pharmingen, San Diego, CA, USA) was used to detect and quantify apoptosis by flow cytometry according to the manufacturer’s instructions. In brief, MCF-7:5C cells (1 × 10^6^ cells/mL) were seeded in 100-mm dishes and cultured overnight in estrogen-free RPMI 1640 medium containing 10% SFS. The next day, cells were treated with <0.1% ethanol vehicle (control) and E_2_ (1 nM) for 96 hours and then harvested in cold PBS (Invitrogen) and collected by centrifugation for 10 minutes at 500 × *g*. Cells were then resuspended at a density of 1 × 10^6^ cells/mL in 1 × binding buffer (HEPES buffer, 10 mM, pH 7.4, 150 mM NaCl, 5 mM KCl, 1 mM MgCl2 and 1.8 mM CaCl2) and stained simultaneously with FITC-labeled annexin V (25 ng/mL; green fluorescence) and propidium iodide (PI) (50 ng/mL). PI was provided as a 50 μg/mL stock (Pharmingen) and was used as a cell viability marker. Cells were analyzed using the BD LSR II flow cytometer (BD Bioscience, San Jose, CA, USA), and the data were analyzed with CellQuest software.

### Immunofluorescence microscopy

Cells grown on glass coverslips were washed in PBS and fixed with 4% formaldehyde in PBS for 30 minutes. After permeabilization by 0.2% Triton X-100 in PBS for 5 minutes, cells were incubated with 0.1 mg/mL RNAse in 2% whole goat serum/PBS for 30 minutes at 37°C, followed by incubation with PLSCR1 (4D2) or IFITM1 antibody, 5 μg/mL in 2% goat serum/PBS (Jackson Immuno Research Labs Inc., West Grove, PA, USA) for one hour. Cells were stained with FITC-conjugated labeled goat anti-mouse IgG, 4 μg/mL in PBS for one hour, followed by nuclear counterstain with 4′,6-diamidino-2-phenylindole (DAPI) 0.1 μg/mL in PBS for 10 minutes. Coverslips were mounted on glass slides with Vectashield Mounting medium (Vector Laboratories, Burlingame, CA, USA), and samples were analyzed on a Bio-Rad MRC-1024 laser scanning confocal microscope equipped with a Zeiss X60 objective. Images were collected using Bio-Rad’s Laser Sharp software. Specificity of staining observed for PLSCR1 mab 4D2 was evaluated by cell staining with the identical concentration of an isotype-matched antibody.

### Cell cycle analysis

MCF-7:5C cells were transfected with siCon, siPLSCR1 or siIFITM1 for 72 hours and then harvested by trypsinization and washed once with phosphate-buffered saline (PBS, pH 7.4). After centrifugation, cells were resuspended in 1 mL of 0.9% NaCl, followed by addition of 2.5 mL of ice-cold 90% ethanol. After incubation at room temperature for 30 minutes, cells were centrifuged and the supernatant was removed. Cells were resuspended in 1 mL PBS containing 50 μg/mL PI and 100 μg/mL ribonuclease A and incubated at 37°C for 30 minutes. Flow cytometric analyses were performed using the BD LSR II flow cytometer (BD Bioscience, San Jose, CA, USA).

### Enzyme-linked immunosorbent assay

Measurement of human interferon-α (IFNα) was conducted by enzyme-linked immunosorbent assay (ELISA) (PBL Interferon Source, Piscataway, NJ, USA). One million MCF-7 or MCF-7:5C cells were seeded in six-well plates and allowed to acclimatize overnight. They were then treated with 250 U/mL human recombinant IFNα for 24 hours. Cells and supernatants were harvested after 24 hours and kept at −80°C until analysis. Protein was extracted by sonication in RIPA buffer supplemented with protease and phosphatase inhibitors. Supernatants and lysates were purified by centrifugation and analyzed for the presence of IFNα according to the manufacturer’s instructions.

### IFNAR neutralization

In order to achieve neutralization of type 1 interferon, IFNAR, MCF-7 and MCF-7:5C cells were pretreated with 5 μg/mL anti-IFNAR1/2/MMHAR2 from Millipore, Temecula, CA, USA (cat# MAB1155) for four hours and then treated overnight with 20 U/mL human recombinant IFNα (Sigma) or 1 nM E_2_ (Sigma) where indicated. Cells were harvested by cell scraping for Western blot and by trypsinization for cell viability analysis with trypan blue count.

### Small interfering RNA transfections

For small interfering RNA (siRNA) knockdown experiments, MCF-7:5C and MCF-7 cells were transiently transfected with PLSCR1-siRNA (h) (siPLSCR1), IFITM1-siRNA (h) (siIFITM1), STAT1-siRNA (h) (siSTAT1), STAT2-siRNA (h) (siSTAT2), IFNα2-siRNA (h) (siIFNα2), IRF-7 siRNA (h) (siIRF-7), Bax-siRNA (h) (siBax), Noxa-siRNA (h) (siNoxa), or nontarget siRNA (siCon). The siPLSCR1 (cat# sc-44028), siIFITM1 (cat# sc-44549), siSTAT1 (cat# sc-44123), siSTAT2 (cat# sc-29492), siIFNα (cat# sc-63324), siIRF-7 (cat# 38011) and siRNA negative control (cat# sc-37007) were purchased from Santa Cruz Biotechnology, and siBax and siNoxa were purchased from Thermo Fisher Scientific (Pittsburg, PA, USA). All of the siRNAs were pools of three target-specific 20 to 25 nt siRNAs. MCF-7:5C or MCF-7 cells were seeded the night before transfection at a density of 30% to 50% confluence by the time of transfection. Twenty nmol of siPLSCR1, siIFITM1, siSTAT1 and siRNA negative control were used for transfection using Lipofectamine 2000 (Invitrogen, San Diego, CA, USA) according to the manufacturer’s instructions. Transfected cells were maintained in culture for two days before harvesting and further analyses. The efficiency of the siRNA knockdown was determined by Western blot analysis.

### Short hairpin RNA (shRNA) knockdown

MCF-7:5C cells were transiently transfected with IFITM1-shRNA plasmid (h) (shIFITM1, cat# sc-44549-SH) or control-shRNA (shControl, cat# sc-108060) plasmid which were purchased from Santa Cruz Biotechnology. IFITM1 shRNA plasmid is a pool of three different shRNA plasmids. sc-44549-SHA: Hairpin sequence: GATCCCACACTTCTCAAACCTTCATTCAAG AGATGAAGGTTTGAGAAGTGTGTTTTT. Corresponding siRNA sequences (sc-44549A): Sense: CACACUUCUCAAACCUUCAtt; Antisense: UGAAGGUUUGAGAAGUGUGtt. sc-44549-SHB: Hairpin sequence: GATCCCTGTGACAGTCTACCATATTTCAAGAGAATA TGGTAGACTGTCACAGTTTTT. Corresponding siRNA sequences (sc-44549B): Sense: CUGUGACAGUCUACCAUAUtt; Antisense: AUAUGGUAGACUGUCACAGtt. sc-44549-SHC: Hairpin sequence: GATCCCTGTCTACAGTGTCATTCATTCAAGAGATGAATGACA CTGTAGACAGTTTTT. Corresponding siRNA sequences (sc-44549C): Sense: CUGUCUACAGUGUCAUUCAtt; Antisense: UGAAUGACACUGUAGACAGtt. MCF-7:5C cells were seeded in six-well plates and at 50% to 70% confluency were transfected with 3 μg of shIFITM1 or shControl plasmid using Lipofectamine 2000 (Invitrogen, San Diego, CA, USA) according to the manufacturer’s instructions. The transfected cells were incubated for 24 or 48 hours and the efficiency of the shRNA knockdown was determined by Western blot analysis and real-time PCR. The knockdown cells were then used for additional experiments.

### Cell migration and invasion assay

Cell migration was measured in a Boyden chamber using Transwell filters obtained from Corning (Cambridge, MA, USA). MCF-7:5C cells (1 × 10^5^) in 0.5 mL serum-free medium were placed in the upper chamber, and the lower chamber was loaded with 0.8 mL medium containing 10% charcoal-stripped FBS. Cells that migrated to the lower surface of the filters were stained with Wright Giemsa solution, and five fields of each well were counted after 24 hours of incubation at 37°C with 5% CO_2_. Three wells were examined for each condition and cell type, and the experiments were repeated in triplicate. Cell invasion assay was performed using a Chemicon Cell Invasion kit (Chemicon International, Temecula, CA, USA) in accordance with the manufacturer’s protocol. Cells (1 × 10^5^/mL) were seeded onto 12-well cell culture chambers using inserts with 8 μM pore size polycarbonate membrane over a thin layer of extracellular matrix (ECM). Following incubation of the plates for 24 hours at 37°C, cells that had invaded through the ECM layer and migrated to the lower surface of the membrane were stained and counted under the microscope in at least ten different fields and photographed.

### RNA isolation and RT-PCR analysis

Total RNA was isolated from cultured cells using the RNeasy® Mini Kit (Qiagen, Venlo, Netherlands) according to the manufacturer’s procedure. First strand cDNA synthesis was performed from 2.5 μg total RNA using Super- Script Reverse Transcriptase (Invitrogen). cDNA was amplified in a 15-μl PCR mixture containing 1 mM dNTPs, 1× PCR buffer, 2.5 mM MgCl2 and 1 U DNA Taq polymerase (Promega, Madison, WI, USA) with 25 pmol of primers specific for human PLSCR1 (sense: 5′-CATTCACCGGGCTCTCTAC-3′; antisense: 5′-GGCAGCTGGGCA ATCTTGCA-3′), IFITM1 (sense: 5′-GGATTTCGGCTTGTCCCGAG-3′; antisense: 5′- CCATG TGGAAGGGAGGGCTC-3′), IRF-9 (sense: 5′-TTCTGTCCCTGGTGTAGAGCCT-3′, antisense: 5′- TTTCAGGACACGATTATCACGG-3′), IRF-7 sense: 5′-GAGCCCTTACCTCCC CTGTTAT-3′, antisense: 5′’-CCACTGCAGCCCCTCATAG-3′, IFI27 (sense: 5′- GCCTCTGG CTCTGCCGTAGTT-3′, antisense: 5′-ATGGAGGACGAGGCGATTCC-3′), IFIT1 (sense 5′-TCTCAGAGGAGCCTGGCTAA-3′, antisense 5′-CCAGACTATCCTTGACCTGATGA-3′), MX1 (sense: 5′-CTTTCCAGTCCAGCTCGGCA-3′, antisense: 5′-AGCTGCTGGCCGTACGT CTG-3′), OAS1 sense: 5′-TGAGGTCCAGGCTCCACGCT-3′, antisense: 5′-GCAGGTC GGTGCACTCCTCG-3′), STAT1 (sense: 5′-GGCACCAGAACGAATGAGGG-3′, antisense: 5′-CCATCGTGCACATGGTGGAG-3′, STAT2 (sense: 5′-GCAGCACAATTTGCGGAA-3′, antisense: 5′-ACAGGTGTTTCGAGAACTGGC-3′). The condition in the logarithmic phase of PCR amplification was as follows: five minutes initial denaturation at 94°C, one minute denaturation at 94°C, 35 seconds annealing at 67°C and 1.5 min extension at 72°C for 30 cycles. The number of amplification cycles during which PCR product formation was limited by template concentration was determined in pilot experiments. PUM1 was used as the internal control (sense: 5′-TCACCGAGGCCCCTCTGAACCCTA-3′; antisense: 5′-GGCAGTAATCTCCTTCTGCATCC T-3′). The reproducibility of the quantitative measurements was evaluated by three independent cDNA syntheses and PCR amplification from each preparation of RNA. Densitometric analysis was performed using Scion Image software (Scion Corp, Frederick, MD, USA), and the relative mRNA expression level was determined as the ratio of the signal intensity of the target to that of PUM1.

### Tissue microarray construction and immunohistochemistry

Paraffin-embedded de-identified human breast cancer tissue samples were collected from the Tumor Bank facility at The Research Institute of Fox Chase Cancer Center (Philadelphia, PA, USA) and the University of Kansas Medical Center (KUMC) and the protocols were reviewed and approved by the Institutional Review Board at Fox Chase Cancer Center and KUMC. The archived tumor samples were collected from patients (N = 40) who were initially treated with Arimidex and either responded or responded but then developed recurrence disease with an average time to disease progression (TTP) of 93 months. Patients provided written informed consent for the use of their tumor samples. Tissue microarray (TMA) slides were constructed from 40 matching primary and AI-resistant tumors using duplicate cores of 0.6 mm per tumor sample. Normal mammary tissue samples (N = 10) were also included on the TMA. For immunohistochemistry assays, tissue microarray slides were incubated at room temperature for 20 minutes with antibodies against IFITM1 (Santa Cruz Biotechnology) and PLSCR1 (Chemicon Inc.) applied at 1:100 dilution in antibody diluent (Dako, Carpenteria, CA, USA). A secondary anti-mouse antibody polymer conjugated with HRP (Dako) was applied for 30 minutes and 3,3′-diaminobenzidine (DAB) was used to produce visible, localized staining viewable with light microscopy. Sections without primary antibody served as negative controls. A semiautomated quantitative image analysis system, ACIS II (ChromaVision Medical Systems, Inc., San Juan Capistrano, CA, USA), was used to quantitate the staining of the TMA slides. For immunohistochemical analysis, the scores were determined by combining the proportion of positively stained tumor cells and the intensity of staining, giving rise to a Staining Index (SI) value for each sample. The proportion of positively stained tumor cells was graded as follows: 0 (<5% positively stained tumor cells), 1 (5% to 25% positive tumor cells), 2 (25% to 50% positive tumor cells), 3 (50% to 75% positive tumor cells) and 4 (>75% positive tumor cells). The intensity of staining was recorded on a scale of 0 (no staining), 1 (weak staining, light brown), 2 (moderate staining, yellowish brown) and 3 (strong staining, brown). The SI value was calculated as follows: SI = staining intensity × proportion of positively stained tumor cells. Scores were evaluated comparatively for the expression of IFITM1 and PLSCR1 in breast tumors by SIs (scored as 0, 1, 2, 3, 4, 6 or 9). An optimal cutoff value was identified, and the SI score of ≥6 was used to define tumors with high expression and SI ≤3 as tumors with low expression of IFITM1 and PLSCR1. Immunohistochemistry (IHC) analysis was also performed on MCF-7, T47D and MCF-7:5C breast cancer cells to detect IFITM1 and PLSCR1 protein expression. Cells were cultured in their appropriate medium, harvested by cell scraper before reaching confluence, washed twice with PBS and fixed in 10% formalin for 16 hours. Each cell line was pelleted and made into a cell block. One H & E stain and two IHC stains for PLSCR1 and IFITM1 were subsequently performed for each cell line. Pretreatments consisted of enzyme digestion or other heat mediated retrieval methods. Sections were stained on a Dako Autostainer using either an Envision PlusHRP polymer (Dako) or horse anti-mouse IgG-biotin (Vector Laboratories, Inc. Burlingame, CA, USA), streptavidin-HRP (Jackson Labs) and AEC (Dako), and counterstained in hematoxylin.

### Statistical analysis

At least three separate experiments were performed for each measurement. All quantitative data were expressed as mean S.D. Comparisons between two groups were analyzed using two-way analysis of variance (ANOVA), with *P* value of <0.05 considered to be statistically significant.

## Results

### IFITM1 and PLSCR1 are constitutively overexpressed in AI-resistant human breast cancer cells and AI-resistant tumors

**Figure 1:**
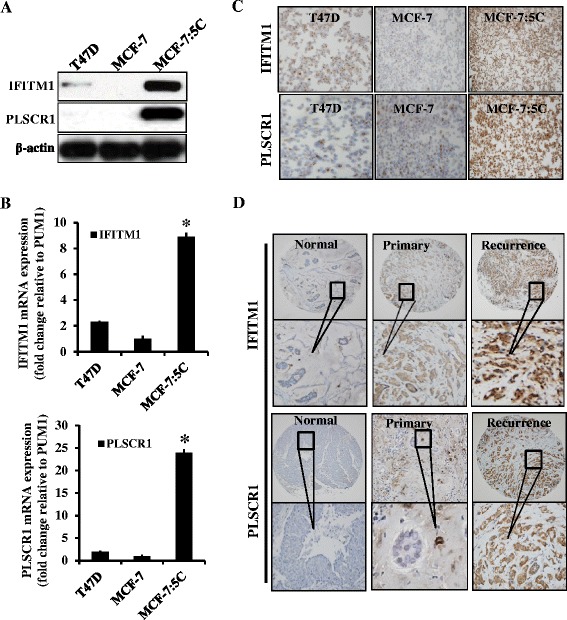
**IFITM1 and PLSCR1 expression in endocrine-sensitive and AI-resistant breast cancer cells. (A)** The cell extracts isolated from the indicated cell lines (endocrine sensitive-T47D/MCF-7, AI-resistant MCF-7:5C) were detected by Western blot analysis for PLSCR1 and IFITM1 protein and β-actin as a loading control. **(B)** Total RNA was extracted from each cell line and IFITM1 (upper panel) and PLSCR1 (lower panel) mRNA was determined by real-time PCR. Fold change was determined for each cell line relative to the internal control gene PUM1. Each value is a mean ± SD from three experiments. *P <0.05 **(C)** Relative intensity of the IFITM1 or PLSCR1 in T47D, MCF-7 and MCF-7:5C cells were determined by immunocytochemistry (ICC) staining. **(D)** Immunohistochemistry (IHC) staining for IFITM1 or PLSCR1 was performed on tissue microarrays generated from normal breast tissue (left panel), primary breast tumor tissue (middle panel) and recurrence breast tumor tissue (right panel). For immunohistochemical analysis, the scores were determined by combining the proportion of positively stained tumor cells and the intensity of staining, giving rise to a Staining Index (SI) value for each sample. The proportion of positively stained tumor cells was graded as follows: 0 (<5% positively stained tumor cells), 1 (5% to 25% positive tumor cells), 2 (25% to 50% positive tumor cells), 3 (50% to 75% positive tumor cells) and 4 (>75% positive tumor cells). Representative photomicrographs were taken using a phase-contrast microscope (original magnification, ×200). AI, aromatase inhibitor; IFITM1, interferon induced transmembrane protein1; PLSCR1, phospholipid scramblase 1; SD, standard deviation.

Microarray studies previously revealed that the interferon signaling pathway was altered in AI-resistant breast cancer cells compared with AI-sensitive cells [17]. To understand better the role of the interferon signaling pathway in AI-resistant breast cancer we measured the basal expression of two well-known interferon stimulated proteins, IFITM1 and PLSCR1, in AI-resistant MCF-7:5C breast cancer cells and AI-sensitive MCF-7 and T47D cells. Our data showed that IFITM1 and PLSCR1 were constitutively overexpressed at the protein (Figure 1A) and mRNA level (Figure 1B) in AI-resistant MCF-7:5C cells but were almost undetectable at the protein and mRNA level in AI-sensitive MCF-7 and T47D cells. Notably, we also found that several other ISGs including IFI27, IFIT1, OAS1, MX1, IRF-7, IRF-9, STAT1 and STAT2 were constitutively overexpressed in AI-resistant MCF-7:5C cells compared with MCF-7 cells (Additional file 1: Figure S1). Immunocytochemistry (ICC) staining of MCF-7, T47D and MCF-7:5C cells also showed that IFITM1 and PLSCR1 were overexpressed in MCF-7:5C cells compared to MCF-7 and T47D cells (Figure 1C). Next, we investigated the clinical significance of IFITM1 and PLSCR1 expression in AI-resistant (recurrence) breast cancer by performing IHC staining on normal breast tissue, primary breast tumors (N = 40) and AI-resistant recurrence breast tumors (N = 40). We found that IFITM1 and PLSCR1 proteins were overexpressed in 90% of the AI-resistant (recurrence) tumors (36 of 40 samples) compared with only 20% of the primary tumors (8 out of 40 samples); however, in normal breast tissue PLSCR1 and IFITM1 proteins were undetectable (Figure 1D). As shown in Table 1, stained slides were scored in terms of intensity and distribution. Normal breast tissue showed no staining for IFITM1 or PLSCR1 (SI score = 0); primary tumors showed medium staining for IFITM1 and PLSCR1 which correlated with low expression (SI score of ≤3); and AI-resistant (recurrence) tumors showed very strong staining for IFITM1 and PLSCR1 which correlated with high expression of both proteins (SI score of ≥6). Taken together, these results demonstrate that interferon regulated genes are constitutively overexpressed in AI resistant breast cancer and they suggest that interferon signaling might be deregulated in the resistant cells.

**Table 1:** **Staining intensity of IFITM1 and PLSCR1 expression in normal and breast tumor tissues**

**Tissue Type**	**Staining**		
	**Intensity**	**IFITM1**	**PLSCR1**
Normal tissues	No stain	10/10	9/10
	Weak	0/10	1/10
	Strong	0/10	0/10
Primary tumors	No stain	32/40	32/40
	Weak	8/40	8/40
	Strong	0/40	0/40
Recurrence tumors	No stain	0/40	0/40
	Weak	4/40	2/40
	Strong	36/40	36/40

Staining intensity was calculated for IFITM1 and PLSCR1 expression in normal breast tissue (N = 10), primary breast tumors (N = 40), and AI-resistant/recurrence breast tumors (N = 40) as described in Methods. AI, aromatase inhibitor; IFITM1, interferon induced transmembrane protein1; PLSCR1, phospholipid scramblase 1.

### IFITM1 and PLSCR1 are localized primarily in the cytoplasm in AI-resistant cells

**Figure 2:**
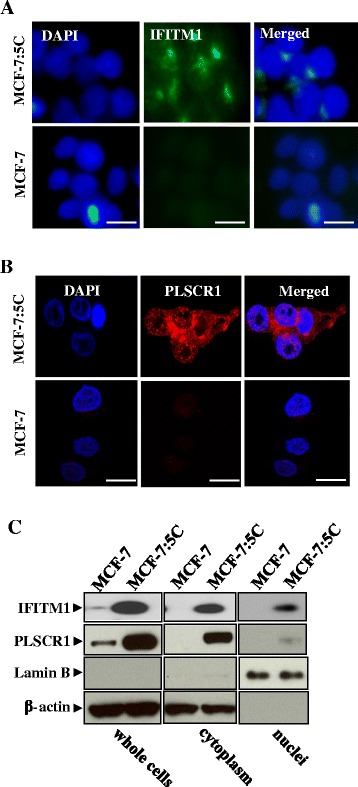
**Subcellular localization of IFITM1 and PLSCR1 in MCF-7 and MCF-7:5C breast cancer cells.** Immunofluorescence staining was performed on parental MCF-7 and AI-resistant MCF-7:5C cells to assess the subcellular localization of **(A)** IFITM1 or **(B)** PLSCR1. Cells were seeded in six-well chamber slides and after 24 hours were analyzed for immunofluorescence staining. **(C)** The whole-cell lysates, nuclear and cytoplasmic/membrane fractions of MCF-7 and MCF-7:5C cells were detected by Western blot analysis for IFITM1 or PLSCR1 protein with β-actin and lamin B as controls. AI, aromatase inhibitor; IFITM1, interferon induced transmembrane protein1; PLSCR1, phospholipid scramblase 1.

Previous studies have shown that IFITM1 and PLSCR1 localize primarily in the plasma membrane [38,39]; however, these proteins can also translocate to the nucleus and bind genomic DNA. We examined the cellular localization of IFITM1 and PLSCR1 in AI-resistant MCF-7:5C cells and AI-sensitive MCF-7 cells using immunofluorescence (IF). Our results showed that IFITM1 (Figure 2A) and PLSCR1 (Figure 2B) were highly expressed in resistant MCF-7:5C cells compared to parental MCF-7 cells and that both proteins localized primarily in the cytoplasm with minor nuclear localization. Western blot analysis of fractionated MCF-7 and MCF-7:5C cells confirmed that IFITM1 and PLSCR1 were overexpressed in MCF-7:5C cells compared with MCF-7 cells and that both proteins were localized primarily in the cytoplasm with some nuclear localization observed for IFITM1 (Figure 2C).

### IFNα drives overexpression of IFITM1 and PLSCR1 in AI-resistant MCF-7:5C cells

**Figure 3:**
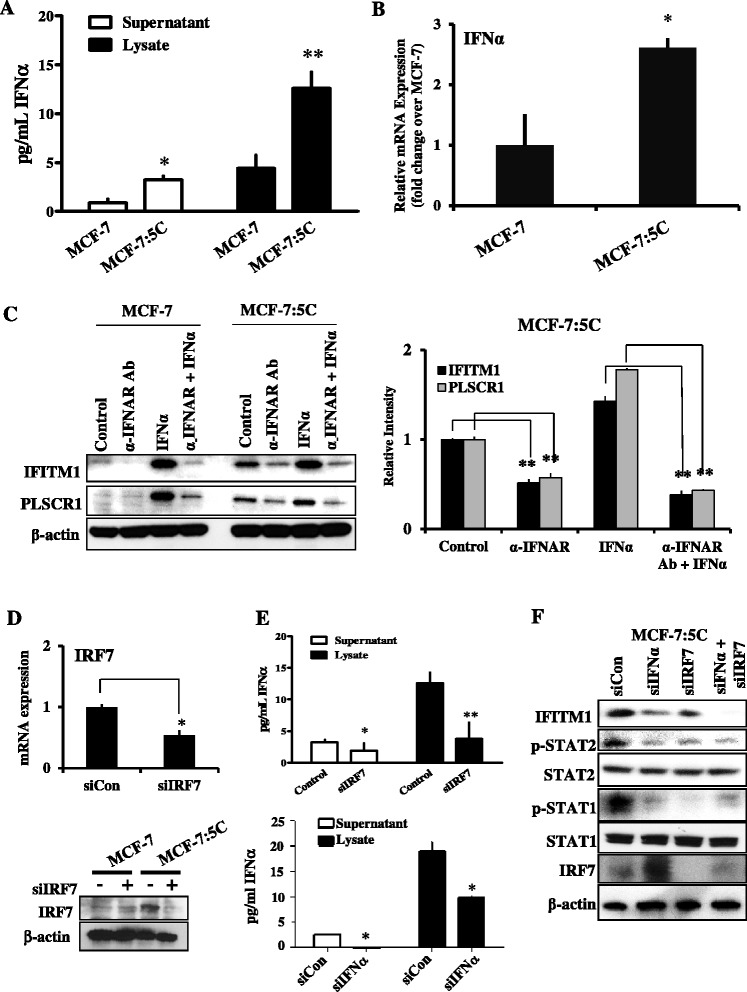
**Elevated level of intracellular IFNα drives constitutive overexpression of IFITM1. (A)** ELISA analysis of baseline expression of IFNα in cell lysates and supernatant in MCF-7:5C and MCF-7 cells. Cells (1 x 10^6^) were seeded in a six-well plate in their standard culture media and after 48 hours cells were harvested and the supernatant and pellets were collected. Cell pellets were then lysed by sonication in RIPA buffer containing protease inhibitors. IFNα was measured in the supernatants and cell lysates by ELISA as described in Methods. All the illustrated data were performed in duplicate and are expressed as mean values of three independent experiments ± SD. **(B)** Measurement of IFNα mRNA was determined by real-time PCR. Fold change was calculated by means of the ΔΔCT method using PUM1 as an internal control. Values are displayed as relative to MCF-7 cells and are means of triplicate measurements ± SD in three independent experiments. **(C)** Blockade of type 1 interferon receptor, IFNAR1, using neutralizing antibody MAB1155, in MCF-7 and MCF-7:5C cells. Cells were pretreated with 5 μg/mL anti-IFNAR1/2 for 4 hours and then treated with 20 U/mL human recombinant IFNα for 24 hours. Cells were analyzed by Western blot to assess IFITM1, PLSCR1 and β-actin protein level. Results shown are representative of three independent experiments. The protein levels were quantified using the ImageJ software (downloaded from NIH website [40]) and normalized as the ratio related to β-actin. *P <0.05 or **P <0.01 versus control. **(D)** siRNA knockdown of IRF-7 expression in MCF-7:5C cells. Cells were transiently transfected with siRNA targeting IRF-7 and after 24 hours knockdown was verified at the mRNA and protein level via RT-PCR and Western blot analyses. **(E)** Effect of IRF-7 knockdown on IFNα expression in resistant MCF-7:5C cells. Cells were transiently transfected with siRNA targeting IRF-7. After 24 hours, IFNα mRNA and IFNα protein expression were determined by real-time PCR and ELISA, respectively. Data shown for RT-PCR is expressed as fold change over cells transfected with control siRNA. Values are displayed as means ± SD of three independent experiments performed in triplicate. *P <0.05 or **P <0.01. **(F)** The effect of IFNα or IRF-7 knockdown on IFITM1, p-STAT2, STAT2, p-STAT1, STAT1, and IRF-7 protein expression in resistant MCF-7:5C cells. Cells were transiently transfected with siIFNα, siIRF-7 or siCon for 48 hours and Western blot analysis was performed on lysates. Results shown are representative of two independent experiments IFITM1, interferon induced transmembrane protein1; IFNAR1, IFN alpha Type 1 receptor; NIH, National Institutes of Health; PLSCR1, phospholipid scramblase 1; SD, standard deviation.

Binding of interferon alpha (IFNα) to the IFN alpha Type 1 receptor (IFNAR1) complex initiates a signaling cascade comprising phosphorylation and dimerization of STAT1/2 molecules followed by their translocation to the nucleus, where they regulate the expression of ISGs. To investigate whether constitutive overexpression of IFITM1 and PLSCR1 in resistant MCF-7:5C cells is driven by the canonical IFNα signaling pathway, we first measured intracellular IFNα level in the supernatant and lysate of AI-resistant MCF-7:5C and parental MCF-7 cells using ELISA. As shown in Figure 3A, IFNα protein level was significantly higher in the supernatant and lysate of resistant MCF-7:5C cells compared to parental MCF-7 cells. IFNα mRNA expression was also significantly elevated in resistant MCF-7:5C cells compared to MCF-7 cells (Figure 3B). Next, we used a neutralizing antibody against the type 1 interferon receptor, IFNAR1/2, to see whether blocking the receptor reduces IFITM1 and PLSCR1 expression in the resistant cells. As shown in Figure 3C, the IFNAR1/2 neutralizing antibody, α-IFNAR-Ab, markedly reduced the basal expression of IFITM1 and PLSCR1 in resistant MCF-7:5C cells and it completely blocked exogenous IFNα induction of IFITM1 and PLSCR1 in parental MCF-7 cells. We further tested whether suppression of the intracellular IFNα level is capable of reducing IFITM1 and PLSCR1 expression in the resistant cells. Induction of IFNα production is primarily controlled at the transcription level by the transcription factor IRF-7, hence, we performed siRNA knockdown of IRF-7 to suppress intracellular IFNα level in the resistant cells. We should note that IRF-7 mRNA (Additional file 1: Figure S1) and IRF-7 protein (Figure 3D, bottom panel) were constitutively overexpressed in resistant MCF-7:5C cells compared to parental MCF-7 cells. As shown in Figure 3D, siIRF-7 markedly reduced IRF-7 mRNA and protein expression in resistant MCF-7:5C cells and it significantly decreased IFNα protein (Figure 3E, top) and IFNα mRNA level (data not shown) in these cells. In addition, we found that siRNA knockdown of IFNα reduced its protein level in the supernatant by 100% and in the lysate by 50% (Figure 3E, bottom). Furthermore, we found that siRNA knockdown of both IFNα and IRF-7 completely reduced IFITM1, PLSCR1, p-STAT1 and p-STAT2 protein expression in the resistant cells (Figure 3F). Taken together, these data indicate that IFNα is significantly elevated in the supernatant and lysate of AI-resistant MCF-7:5C breast cancer cells and that activation of the canonical IFNα/IFNAR signaling pathway plays a critical role in driving the constitutive overexpression of IFITM1 and other ISGs in the resistant cells.

### Dysregulation of type 1 IFNα signaling in AI-resistant MCF-7:5C cells

**Figure 4:**
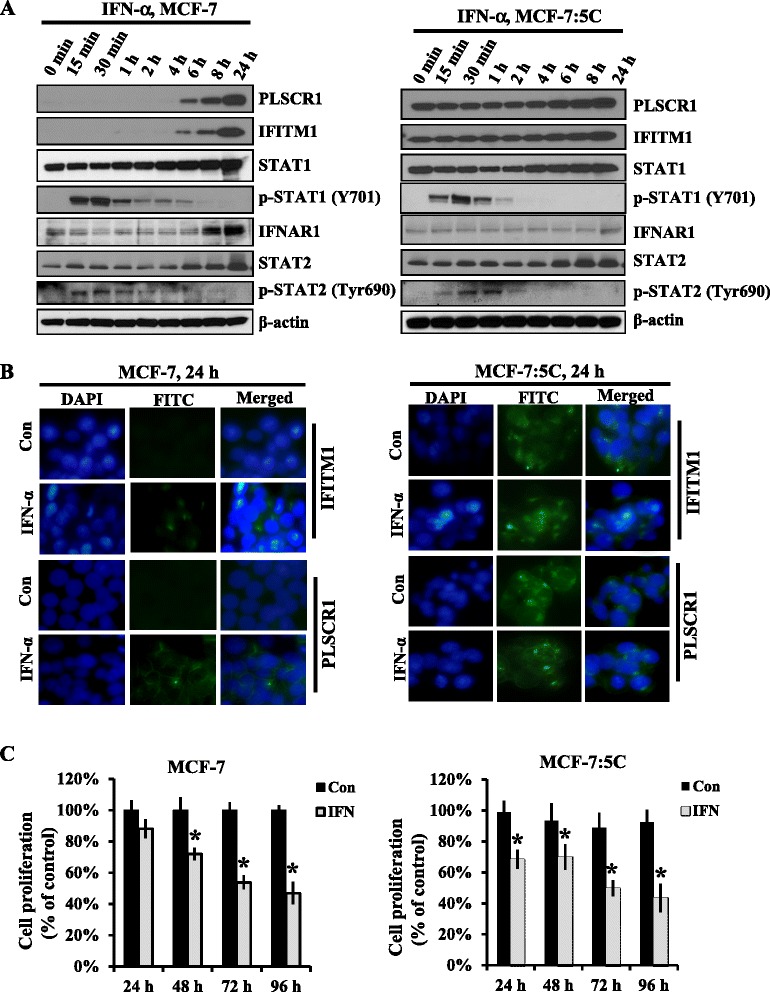
**Activation of interferon signaling pathway in parental MCF-7 and AI-resistant MCF-7:5C cells in response to INFα. (A)** MCF-7 (left panel) and MCF-7:5C (right panel) cells were incubated with IFNα (1000 U/ml) for the indicated time points. The cell extracts were examined by Western blotting using anti-PLSCR1, anti-IFITM1, anti-STAT1, anti-phospho-STAT1 (Y701), anti-IFNAR1, anti-STAT2, anti-phospho-STAT2 (Tyr690) and anti-β-actin. **(B)** Cellular localization of IFITM1 and PLSCR1 in MCF-7 and MCF-7:5C cells following IFNα treatment. Cells were treated for 24 hours with IFNα (1000 U/ml) and then analyzed for immunofluorescence staining of IFITM1 and PLSCR1 (original magnification 200×). **(C)** MCF-7 and MCF-7:5C cells were treated with IFNα (1000 U/ml) for 24, 48, 72 and 96 hours and cell proliferation was measured by the MTT assay. All the illustrated data are expressed as mean values of three independent experiments. Standard deviations are shown. *P <0.05; **P <0.01. AI, aromatase inhibitor; IFITM1, interferon induced transmembrane protein1; IFNAR1, IFN alpha Type 1 receptor; MTT, 3-(4,5-dimethylthiazol-2-Yl)-2,5-diphenyltetrazolium bromide; PLSCR1, phospholipid scramblase 1; STAT1,2, Signal transducer and activator of transcription 1,2.

Since IFITM1 and PLSCR1 were constitutively overexpressed in the resistant cells, we wanted to assess the functional integrity of the interferon signaling pathway in the resistant cells compared to parental MCF-7 cells. Cells were treated with 1,000 U/ml of IFNα for 0 to 24 hours and protein levels of IFITM1, PLSCR1, STAT1, p-STAT1 (Y701), STAT2, p-STAT2 (Tyr690) and IFNAR1 were determined by Western blot analysis. We found that in parental MCF-7 cells, IFNα treatment significantly increased PLSCR1, IFITM1, STAT1, p-STAT1, STAT2, p-STAT2 and IFNAR1 protein expression in a time-dependent manner with maximum induction of PLSCR1, IFITM1, STAT1, STAT2 and IFNAR1 observed at 24 hours and for p-STAT1 and p-STAT2 at 30 minutes (Figure 4A, left panel). In contrast, we found that IFITM1 and PLSCR1 were constitutively overexpressed in resistant MCF-7:5C cells and that treatment with exogenous IFNα only increased p-STAT and p-STAT2 protein; however, it did not further increase the level of IFITM1, PLSCR1 or STAT1 at any of the time points except at 24 hours where we detected a <2-fold increase in PLSCR1 and IFITM1 (Figure 4A, right panel; Additional file 2: Figure S2). A similar trend was observed at the mRNA level for IFITM1, PLSCR1 and STAT1 in resistant MCF-7:5C cells compared to parental MCF-7 cells (Additional file 3: Figure S3). In MCF-7 cells, exogenous IFNα induced IFITM1 mRNA by approximately 374-fold, PLSCR1 mRNA by approximately 9-fold and STAT1 mRNA by approximately 11-fold at 48 hours, whereas, in resistant MCF-7:5C cells, treatment with IFN-α induced IFITM1 mRNA by approximately 3-fold, PLSCR1 mRNA by approximately 2-fold, and STAT1 mRNA by approximately 2-fold (Additional file 3: Figure S3). These findings suggest that IFITM1, PLSCR1 and STAT1 are constitutively overexpressed in the resistant cells due to dysregulation of interferon signaling, whereas, in parental MCF-7 cells, the interferon signaling pathway is functionally intact and the induction of IFITM1 and other ISGs is tightly controlled.

Next, we examined whether IFNα treatment facilitated translocation of IFITM1 and PLSCR1 from the cytoplasm to the nucleus. As shown in Figure 4B, treatment with IFNα significantly increased IFITM1 and PLSCR1 protein expression in MCF-7 cells but not in AI-resistant MCF-7:5C cells; however, it did not cause IFITM1 or PLSCR1 to translocate from the cytoplasm to the nucleus in either cell line. Interestingly, cell viability assay showed that IFNα treatment significantly inhibited the proliferation of both MCF-7 and MCF-7:5C cells in a time-dependent manner (Figure 4C) and that AI-resistant MCF-7:5C cells were slightly more sensitive to the growth inhibitory effect of IFNα than MCF-7 cells.

### Knockdown of IFITM1 induces cell death in AI-resistant MCF-7:5C breast cancer cells

**Figure 5:**
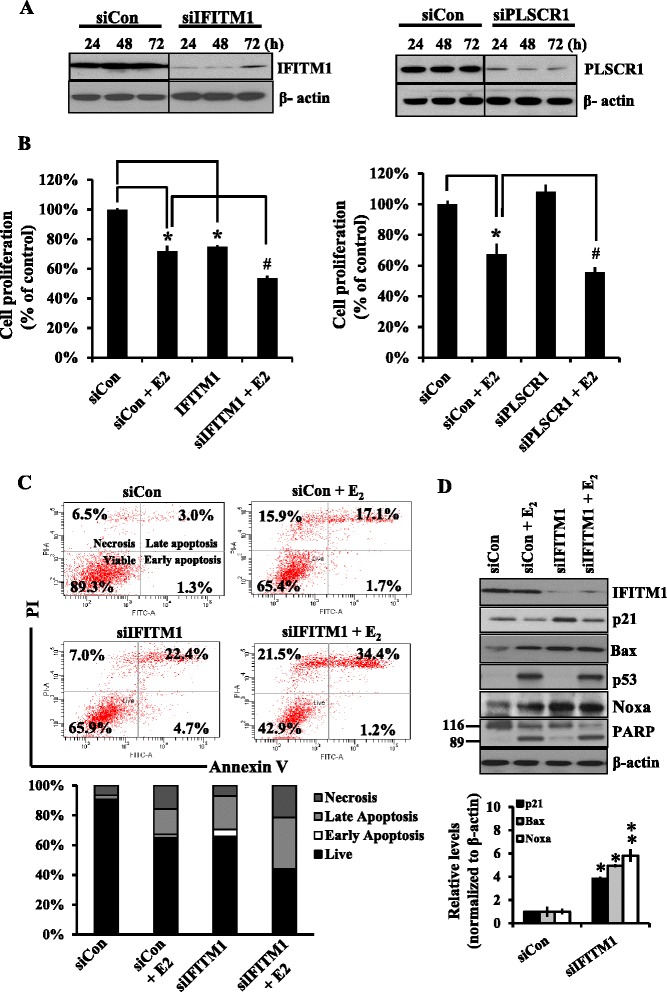
**IFITM1 knockdown increases cell death in AI-resistant breast cancer cells. (A)** MCF-7:5C cells were transfected with control siRNA (siCon), IFITM1 siRNA (siIFITM1) or PLSCR1 siRNA (siPLSCR1) for 24, 48 and 72 hours and cell extracts were subject to Western blotting analysis to assess IFITM1 and PLSCR1 protein expression. **(B)** MCF-7:5C cells were transfected with siCon, siIFITM1 (left panel) or siPLSCR1 (right panel) for 24 hours and then treated with 1 nM E_2_ for an additional 72 hours. Cell proliferation was measured by the MTT assay. All the illustrated data are expressed as mean values of three independent experiments. Standard deviations are shown. *P <0.05 versus control; #*P* <0.05 versus E_2_ treatment. **(C)** MCF-7:5C cells were transfected with siCon or siIFITM1 and after 24 hours were exposed to E_2_ (1 nM) for an additional 96 hours. Cells were then stained with annexin V-FITC and PI for detection of apoptosis as described in Methods. **(D)** Cells were treated as described above in **(C)** and were analyzed by Western blotting to assess IFITM1, p21, Bax, p53, Noxa and PARP protein expression. Membranes were stripped and reprobed for β-actin, which was used as a loading control. The protein levels for p21, Bax and Noxa were quantified using the ImageJ software (downloaded from the NIH website) and normalized as the ratio relate to β-actin. *P <0.05; **P <0.01. AI, aromatase inhibitor; E_2_, 17β-estradiol; FITC, fluorescein isothiocyanate; IFITM1, interferon induced transmembrane protein1; IFNAR1, IFN alpha Type 1 receptor; MTT, 3-(4,5-dimethylthiazol-2-Yl)-2,5-diphenyltetrazolium bromide; NIH, National Institutes of Health; PARP, poly ADP ribose polymerase; PI, propidium iodide; PLSCR1, phospholipid scramblase 1.

Previous studies have reported that IFITM1 and PLSCR1 exert both antiproliferative and proliferative effects in different types of cancer cells; however, their functional significance in AI-resistant breast cancer cells is not known. To determine the functional significance of IFITM1 and PLSCR1 in AI-resistant MCF-7:5C breast cancer cells, we transiently transfected MCF-7:5C cells with siRNA targeting IFITM1 or PLSCR1 and we assessed the effect of their knockdown on cell proliferation, cell death and cell cycle progression. Western blot analysis confirmed knockdown of IFITM1 and PLSCR1 protein in AI-resistant MCF-7:5C cells at 24, 48 and 72 hours post-transfection (Figure 5A). Cell viability assay showed that knockdown of IFITM1, but not PLSCR1, significantly inhibited the proliferation of AI-resistant MCF-7:5C cells at 72 hours relative to control cells and it markedly enhanced the inhibitory effect of E_2_ in these cells (Figure 5B). The growth inhibitory effect of IFITM1-knockdown in AI-resistant MCF-7:5C cells was due to cell death, as demonstrated by annexin V-PI staining (Figure 5C). Specifically, knockdown of IFITM1 in MCF-7:5C cells increased the total number of dead cells from 10.8% (siCon) to 35.1% in the IFITM1-knockdown cells and it enhanced the apoptotic effect of E_2_ from 34.6% to 57.1% (Figure 5C). We should note that the ability of E_2_ to induce cell death in AI-resistant breast cancer cells has previously been reported by our laboratory [11,15,16]; however, this is the first study to show that suppression of IFITM1 enhances E_2_-induced cell death in AI resistant breast cancer cells. Further analysis indicated that knockdown of IFITM1 significantly increased the expression of p21, Bax and Noxa in AI-resistant MCF-7:5C cells; however, it did not significantly alter p53 expression in these cells (Figure 5D). To validate the specificity and the biological function of IFITM1 in our resistant cells we used a second shRNA targeting IFITM1 (Additional file 4: Figure S4). We found that shRNA knockdown of IFITM1 induced poly ADP ribose polymerase (PARP) cleavage (Additional file 4: Figure S4A), reduced cell proliferation (Additional file 4: Figure S4B) and induced cell death in resistant MCF-7:5C cells which was further enhanced by the addition of E_2_ (Additional file 4: Figure S4C). To confirm that IFNα was responsible for the dysregulation of IFITM1 and that blocking its function enhances E_2_-induced cell death, we knockeddown IFNα expression in resistant MCF-7:5C cells and then treated the cells with E_2_ for an additional 96 hours. As shown in Additional file 5: Figure S5, knockdown of IFNα significantly reduced the proliferation of MCF-7:5C cells and it significantly enhanced E_2_-induced death in these cells at 96 hours. Furthermore, we found that blocking IFNAR1/2 with a neutralizing antibody also reduced the proliferation of MCF-7:5C cells and it markedly enhanced E_2_-induced death in these cells at the same time point. These findings confirm that IFNα is responsible for the dysregulated expression of IFITM1 in the resistant cells and that blocking its function collaborates with E2 to enhance cell death in these cells. Furthermore, these findings suggest that IFITM1 overexpression provides a survival advantage to the resistant cells that allows them to grow in an estrogen-depleted environment and that knockdown of IFITM1 disrupts the survival pathway in these cells thus sensitizing them to cell death.

### IFITM1 knockdown inhibits migration and invasion of AI-resistant MCF-7:5C cells

**Figure 6:**
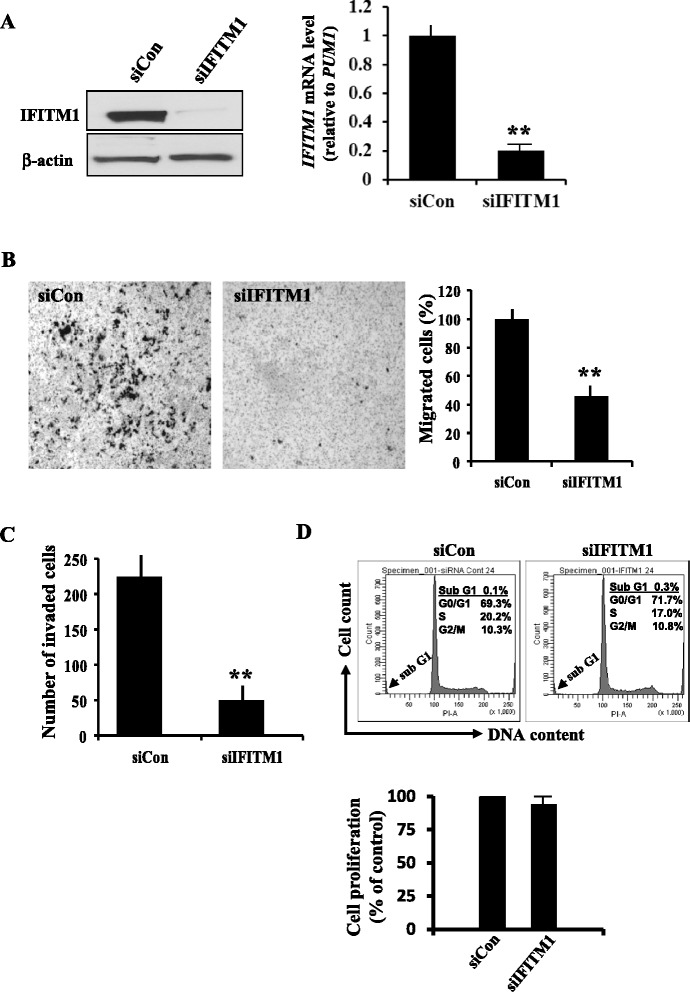
**IFITM1 knockdown decreases migration and invasion in AI-resistant MCF-7:5C breast cancer cells. (A)** MCF-7:5C cells were transfected with control siRNA (siCon) or IFITM1 siRNA (siIFITM1) for 24 hours and knockdown of IFITM1 protein expression was confirmed by Western blot (right panel) and real-time PCR analyses (left panel). Standard deviations are shown. **P <0.01 versus siCon. **(B and C)** The effect of IFITM1 knockdown on cell migration **(B)** and invasion **(C)** was assessed by transwell migration assay and matrigel invasion assay. Cells that invaded through the Matrigel-coated transwells were fixed, stained, visualized by light microscopy and photographed. Quantitation of the Transwell assay is also shown (**B**, right panel). Ten random fields were counted per insert at 20X. **P <0.01. **(D)** Effect of IFITM1 knockdown on cell viability in resistant MCF-7:5C cells. Cells were transfected with siCon or siIFITM1 for 24 hours, then DNA content of cells was analyzed using flow cytometry as described in the Methods section. The arrow is sub G1 phase apoptosis. MTT assay (bottom panel) was also performed in the IFITM1-knockdown cells at 24 hours. All the illustrated data are expressed as mean values of three independent experiments. Standard deviations are shown. AI, aromatase inhibitor; IFITM1, interferon induced transmembrane protein1; MTT, 3-(4,5-dimethylthiazol-2-Yl)-2,5-diphenyltetrazolium bromide.

There is evidence that IFITM1 overexpression induces tumor resistance to natural killer (NK) cells in gastric tumor cells and it facilitates migration and invasion of gastric cancer cells [29]. In addition, overexpression of IFITM1 has been shown to promote head and neck tumor invasion by mediating the expression of matrix metalloproteinases 12 and 13 [32]. To investigate the role of IFITM1 in breast cancer progression, we examined the influence of IFITM1 knockdown on migration and invasion of AI-resistant MCF-7:5C breast cancer cells. Western blot and real-time PCR analysis confirmed that IFITM1 protein and mRNA expression suppressed by siRNA in MCF-7:5C cells compared with siCon-transfected cells (Figure 6A). Silencing of IFITM1 markedly reduced the migratory ability (Figure 6B) and invasion capacity (Figure 6C) of AI-resistant MCF-7:5C cells. The cell migration and invasion counted from 10 randomly selected areas per well at 24 hours showed that siRNA knockdown of IFITM1 inhibited migration by 54% (Figure 6B, bar graph) and invasion by approximately 78% (Figure 6C) compared with siCon-transfected cells. To confirm that the inhibitory effect of IFITM1 knockdown on migration and invasion was not due to cell death we measured apoptosis (via flow cytometry) and cell viability in IFITM1-knockdown MCF-7:5C cells at the same time point (24 hours) the migration and invasion assays were performed. As shown in Figure 6D, IFITM1 knockdown did not induce cell death (top panel) or reduce cell viability (bottom panel) at 24 hours; hence, its inhibitory effect on migration and invasion at 24 hours is not due to cell death. However, we should note that knockdown of IFITM1 does cause significant cell death at 72 hours; hence, migration and invasion would be inhibited at the later time points due to cell death. This result suggests that overexpression of IFITM1 enhances the ability of AI-resistant MCF-7:5C cells to migrate and invade and its suppression has the opposite effect.

### STAT1 and STAT2 regulate IFITM1 and PLSCR1 expression in resistant MCF-7:5C cells

**Figure 7:**
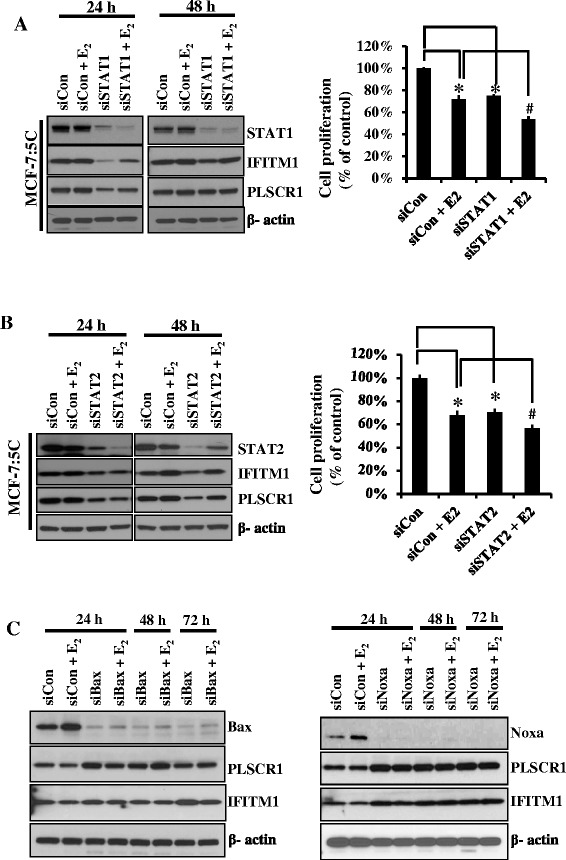
**STAT1/STAT2 knockdown reduces IFITM1 and PLSCR1 expression. (A and B)** MCF-7:5C cells were transfected with sicontrol (siCon), STAT1 siRNA (siSTAT1) or STAT2 siRNA (siSTAT2) and STAT1, STAT2, IFITM1 and PLSCR1 protein levels were assessed at 24 and 48 hours by Western blot analysis (left panels). Transfected cells were also treated with E_2_ for an additional 24 and 48 hours and the above mentioned proteins were also measured (a and b, left panels). **(A, B)** Cell proliferation was measured in siSTAT1-knockdown and STAT2-knockdown cells in the presence or absence of E_2_ by cell titer blue assay (right panels). Each value is a mean ± SD from three experiments. *P <0.05 or **P <0.01 versus the control; #*P* <0.05 versus E_2_ treatment. **(C)** MCF-7:5C cells were transfected with Bax siRNA (siBax) or Noxa siRNA (siNoxa) for 24 hours and then treated with 1 nM E_2_ for an additional 24, 48 or 72 hours. Cells were harvested and analyzed for Bax, PLSCR1, Noxa, and IFITM1 protein expression by Western blot. Membranes were stripped and reprobed for β-actin, which was used as a loading control. E_2_, 17β-estradiol; IFITM1, interferon induced transmembrane protein1; PLSCR1, phospholipid scramblase 1; SD, standard deviation; STAT1,2, Signal transducer and activator of transcription 1,2.

STAT1 and STAT2 are members of the signal transducers and activators of transcription family of transcription factors that play a pivotal role in regulating type I (α/β) and type II (γ) interferon signaling. In response to either IFNα or IFNβ stimulation, STAT1 and STAT2 form homodimers or heterodimers, move to the nucleus and activate the transcription of interferon response genes. Since IFITM1 and PLSCR1 are constitutively overexpressed in AI-resistant MCF-7:5C cells, we examined whether knockdown of STAT1 or STAT2 is capable of altering their expression in MCF-7:5C cells. AI-resistant MCF-7:5C cells were transiently transfected with control siRNA (siCon), STAT1 siRNA (siSTAT1) or STAT2 siRNA (siSTAT2) and the effect of knockdown on IFITM1 and PLSCR1 expression was assessed at 24 and 48 hours using Western blot analysis. As shown in Figure 7A (left panel), knockdown of STAT1 markedly reduced IFITM1 and PLSCR1 protein expression in resistant MCF-7:5C cells at 24 and 48 hours, and it significantly reduced the proliferation of MCF-7:5C cells, and it further enhanced the inhibitory effect of E_2_ in these cells. STAT2 knockdown also reduced IFITM1 and PLSCR1 protein level in MCF-7:5C cells (Figure 7B, left panel), and it significantly enhanced the inhibitory effect of E_2_ in these cells (Figure 7B, right panel). Furthermore, we found that knockdown of both STAT1 and STAT2 completely suppressed IFITM1 and PLSCR1 expression in resistant MCF-7:5C cells (Additional file 6: Figure S6), thereby confirming a critical role for STAT1 and STAT2 in the regulation of IFITM1 and PLSCR1 expression. Interestingly, we found that knockdown of proapoptotic Bax and Noxa enhanced IFITM1 and PLSCR1 expression in MCF-7:5C cells, however, the mechanism by which this occurs is currently not known (Figure 7C).

### 4-Hydroxytamoxifen inhibits IFITM1 expression in AI-resistant MCF-7:5C cells

**Figure 8:**
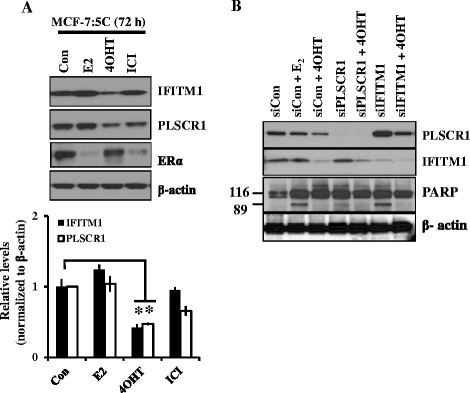
**Tamoxifen downregulates IFITM1 and PLSCR1 expression.** (**A**, upper panel) MCF-7:5C cells were treated with E_2_ (1 nM), 4OHT (1 μM) or fulvestrant (1 μM) for 72 hours. The cell extracts were examined by Western blotting using anti-PLSCR1, anti-IFITM1, anti-ERα and anti-β-actin. (**A**, lower panel) IFITM1 and PLSCR1 protein levels were quantified using the ImageJ software (downloaded from the NIH website) and normalized to β-actin. **(B)** Cells were transfected with siCon, siPLSCR1 or siIFITM1 for 24 hours and then treated with 1 nM E_2_ or 1 μM 4-hydroxytamoxifen (4OHT) for an additional 24 hours. Cells were then harvested and analyzed by Western blotting to assess PLSCR1, IFITM1, PARP and β-actin protein levels. All the illustrated data are expressed as mean values of three independent experiments. Standard deviations are shown. *P <0.05 versus control; #*P* <0.05 versus 4OHT treatment. E_2_, 17β-estradiol; ERα, estrogen receptor α; IFITM1, interferon induced transmembrane protein1; NIH, National Institutes of Health; PARP, poly ADP ribose polymerase; PLSCR1, phospholipid scramblase 1.

Since knockdown of IFITM1 significantly enhanced E_2_-induced cell death in AI-resistant MCF-7:5C cells, we next determined whether IFITM1 expression is regulated by the estrogen receptor (ERα). MCF-7:5C cells were treated with 1 nM E_2_, 1 μM of 4-hydroxytamoxifen (4OHT), the active metabolite to tamoxifen, or 1 μM of fulvestrant (ICI 182,780), the pure antiestrogen that downregulates ERα and the expression of IFITM1, PLSCR1 and ERα were measured by Western blot analysis. As shown in Figure 8A, E_2_ and fulvestrant completely down-regulated ERα protein but did not significantly alter IFITM1 or PLSCR1 expression, whereas 4OHT did not down-regulate ERα protein but it significantly reduced IFITM1 and PLSCR1 expression in the resistant cells. Interestingly, we found that 4OHT also partially blocked the cell death effect of IFITM1-knockdown in MCF-7:5C cells, as demonstrated by inhibition of PARP cleavage (Figure 8B). Thus, it is possible that IFITM1 expression might be regulated by ERα; however; we do not rule out the possibility that 4OHT might be exerting an effect on IFITM1 that is independent of ERα. Further studies are needed to understand better the mechanism by which 4OHT regulates IFITM1 and whether ERα is involved in the process.

## Discussion

Estrogen deprivation by treatment with AIs is the most effective form of endocrine therapy for postmenopausal women with estrogen receptor–positive (ER+) breast cancer. Unfortunately, the majority of patients treated with AIs eventually develop resistance. While the mechanism by which endocrine resistance occurs is still not completely known, there is evidence to suggest that dysregulation in the interferon signaling pathway might play a role in the process. Indeed, studies have reported a strong correlation between constitutive expression of ISGs and resistance to radiotherapy and chemotherapy in several types of human cancers including breast cancer, ovarian cancer, lung cancer and lymphatic leukemia [41-44]. Notably, Dunbier and coworkers [45] recently reported on the poor anti-proliferative response to AI treatment in postmenopausal patients whose tumors expressed high baseline expression of immune response genes; however, these investigators did not examine interferon signaling in AI-resistant breast tumors. In the present study, we have demonstrated that interferon signaling is dysregulated in AI-resistant breast cancer cells and AI-resistant/recurrence tumors and that several interferon response genes including; *IFITM1, PLSCR1, STAT1, STAT2, IRF-7, IRF-9, IFIT1, OAS1* and *MX1* are constitutively overexpressed in AI-resistant breast cancer cells. In particular, we showed that IFITM1 and PLSCR1 were overexpressed at the mRNA and protein level in AI-resistant MCF-7:5C cells compared with AI-sensitive MCF-7 and T47D cells and that suppression of IFITM1, and to a lesser extent PLSCR1, significantly inhibits the proliferation of AI-resistant MCF-7:5C cells and blocked the ability of these cells to invade and migrate. Most interestingly, we found that silencing of IFITM1, PLSCR1 and STAT1 significantly enhanced E_2_-induced cell death in AI-resistant MCF-7:5C cells which was associated with induction of p21, Bax, and Noxa. Additionally, we found that constitutive overexpression of IFITM1 and PLSCR1 was driven by IFNα signaling through the canonical IFNAR1/2/STAT1/STAT2 signaling pathway and that knockdown of STAT1 and STAT2 and blockade of IFNα function dramatically suppressed IFITM1 and PLSCR1 expression in the resistant cells. To our knowledge, this is the first study to demonstrate a link between dysregulation of the interferon signaling pathway and AI resistance and it suggests that targeting IFITM1 might be an effective strategy to block cell proliferation and enhance E_2_-induced cell death in AI-resistant breast cancer cells.

**Figure 9:**
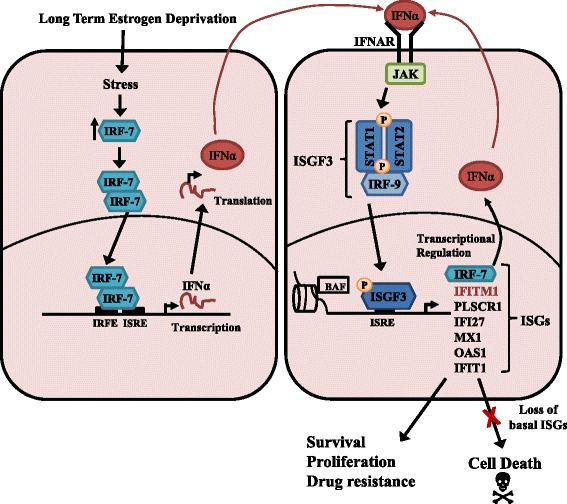
**Schematic diagram depicting the proposed mechanism of ISG expression in AI-resistant MCF-7:5C cells.** ER-positive breast cancers are dependent on estrogen for survival and growth and when these cancer cells are deprived of estrogen they tend to die. Long term, however, some breast cancer cells develop strategies to allow them to survive and grow in an estrogen-depleted environment. In our working model, we propose that long term estrogen deprivation of ERα positive breast cancer cells elicits a stress response in the cell which can possibly result in increased expression and activation of IRF-7, a known stress response gene. The activated IRF-7 enters the nucleus and binds the IFNα promoter at the IRF binding-element (IRFE) and ISRE sites, resulting in IFNα production and secretion from the cell. IFNα then binds to its cell surface receptor IFNAR1/2 which initiates JAK phosphorylation of the STAT proteins and association with IRF-9, forming the activated ISGF3 complex (p-ISGF3). The BAF complex remodels the chromatin around prospective ISGs and p-ISGF3 is imported into the nucleus where it binds to the exposed ISRE sites in ISG promoters. This results in the sustained/constitutive overexpression of numerous ISGs including IFITM1 and IRF-7, which initiates further IFNα production and thus provides autocrine cytokine signaling, reinforcing the production and accumulation of ISGs. The ISGs are pro-survival and so facilitate cell survival and proliferation under the stressful (estrogen depleted) conditions. Loss of expression of the ISGs reduces the ability of the resistant cells to survive in an estrogen-depleted environment thus causing them to die. AI, aromatase inhibitor; ER, estrogen receptor; IFITM1, interferon induced transmembrane protein1; IRF-7, IFN regulatory factor 7; ISGs, interferon stimulated genes; ISREs, IFN-stimulated response elements; JAK, Janus kinase 1 and 2; STAT, Signal transducer and activator of transcription.

Type I interferons (IFNs α and β) are known to drive the expression of ISGs that encode proteins that possess anti-viral, anti-proliferative, pro-apoptotic and pro-inflammatory functions; however, many experimental data have shown that high expression of IFN-induced genes, including STAT1 itself, promotes tumor growth, metastasis and resistance to chemotherapy and radiation [23,44,46,47]. Normally, IFNs induce rapid activation of STATs through phosphorylation on the C-terminal tyrosine residues (Y701 for STAT1 and Y690 for STAT2) which drives the expression of ISGs [19]. Several important negative feedback mechanisms collaborate to terminate the expression of these genes several hours after IFN stimulation; for example, expression of the potent negative regulator SOCS1 is rapidly induced by IFNs [48]. Based on our studies, we observed several ISGs including IFITM1, PLSCR1, IFIT1, IFI23, IRF-7, IRF-9, STAT1, STAT2, MX1 and OAS1 were overexpressed approximately 10- to 300-fold in AI-resistant MCF-7:5C cells compared to AI-sensitive parental MCF-7 cells (Additional file 1: Figure S1). Furthermore, we found that in parental MCF-7 cells, exogenous addition of recombinant IFNα robustly induced the expression of IFITM1, PLSCR1 and STAT1 (10- to 200-fold) within minutes to hours, whereas, in resistant MCF-7:5C cells, exogenous IFNα only increased IFITM1, PLSCR1, and STAT1 expression by 2- to 3-fold compared to basal level (Figure 4A, Fig. S2, Fig. S3). This finding suggests that in parental MCF-7 cells, IFITM1 and other ISGs, are not expressed at the basal level unless stimulated by exogenous IFNα; however, in AI-resistant MCF-7:5C cells, IFITM1, PLSCR1 and other ISGs are constitutively overexpressed possibly due to an elevated level of IFNα in the cells. Notably, we detected significantly elevated levels of IFNα in the supernatant and lysate of AI-resistant MCF-7:5C cells compared to parental MCF-7 cells and knockdown of IFNα along with neutralizing antibody blockade of the receptor (IFNAR1/2) almost completely reduced IFITM1, PLSCR1, p-STAT1 and p-STAT2 expression in the resistant cells, thus suggesting a critical role for the IFNα canonical signaling pathway in driving the constitutive expression of the ISGs in the resistant cells. Sustained expression of ISGs and their encoded proteins was previously thought to be deleterious to cell survival [19]; however, recent studies suggest that sustained expression of a subset of ISGs and their encoded proteins might provide a survival advantage to cells [49]. We should note that ER-positive breast cancer cells are dependent on estrogen for survival and growth and when they are deprived of estrogen they tend to die. Long term, however, some breast cancer cells develop strategies to allow them to survive and grow in an estrogen-depleted environment [10-12,15,16]. In our working model shown in Figure 9, we propose that in AI-resistant MCF-7:5C breast cancer cells, the transcription factor IRF-7 which is a key regulator of IFNα, is dramatically upregulated and that increased IRF-7 expression in the resistant cells stimulates the production of IFNα which is then secreted from the cells and binds to the IFNAR1/2STAT1/STAT2/IRF-9 complex to induce the expression of ISGs (that is, *IFITM1, PLSCR1, IFIT1, IFI21, OAS1, MX1, STAT, STAT2, IRF-7, IRF-9*). Thus, it is possible that the constitutive overexpression of the ISGs provides a survival advantage to the resistant cells that allows them to adapt and grow in an estrogen-depleted environment. The fact that knockdown of IFNα dramatically reduced IFITM1 expression in the resistant cells and its loss significantly induced cell death highlights the potential dependency of the resistant cells on elevated IFNα to maintain their resistant phenotype and to drive the constitutive overexpression of IFITM1 and the other ISGs in the cells. We should note that elevated IFN production has previously been reported in many pathological conditions, such as chronic inflammation and cancer, as well as in virus infections [50]. In cancers, IFN production is thought to be increased by infiltrating immune cells or by the cancer cells themselves [51].

While our studies identified a panel of ISGs that were constitutively overexpressed in our AI-resistant breast cancer cells, IFITM1 was the most functionally significant of the ISGs in the resistant cells. Clinical data indicated that IFITM1 was constitutively overexpressed in 36 out of 40 AI-resistant breast tumor samples and it was overexpressed in AI-resistant breast cancer cells approximately 20- to 30-fold at the mRNA and protein level. Notably, we found that knockdown of IFITM1 significantly increased cell death and it blocked invasion and migration in the resistant cells. The induction of cell death in the resistant cells following IFITM1 knockdown was associated with an increase in p21 and Bax expression; however, suppression of IFITM1 did not alter p53 expression or cause cell cycle arrest in these cells. p21 (also known as p21^WAF1/Cip1^) is a multifunctional protein that belongs to the cip/kip family of cyclin-dependent kinase inhibitors and is known to promote G1/S or G2/M cell cycle arrest and survival in a p53-dependent and p53-independent manner [52]. In addition, p21 has also been shown to play a role in apoptosis and it is suggested that its ability to regulate cell cycle arrest versus apoptosis is influenced by its cellular localization; nucleus accumulation promotes cell cycle arrest whereas cytoplasmic accumulation inhibits apoptosis [53,54]. Notably, suppression of IFITM1 also induced Bax and Noxa which are important regulators of mitochondrial-mediated cell death. Interestingly, previous studies have reported that p21 counteracts mitochondrial-mediated apoptosis that relies on Bax and its upstream effector Puma [55]; however, in our study, p21 induction positively correlated with Bax and Noxa induction thus suggesting a pro-apoptotic function for p21 in the resistant cells. We should note that knockdown of IFITM1 also increased E_2_-induced cell death in AI-resistant MCF-7:5C cells. The ability of E_2_ to induce mitochondrial-mediated apoptosis in AI-resistant MCF-7:5C cells has previously been reported by our laboratory [11,17]; however, this is the first study to show that suppression of IFITM1 induces p21 and Bax expression and enhances E_2_-induced cell death in AI-resistant breast cancer cells.

IFITM1 is a member of the IFN-inducible transmembrane (IFITM) protein family that was originally identified as Leu-13, a leukocyte antigen that is a part of a membrane complex involved in the transduction of antiproliferative and homotypic adhesion signals in lymphocytes [56,57]. IFITM1 is highly induced by type 1 interferons (IFNs α and β) and is most well-known for its ability to restrict the replication of a number of enveloped and non-enveloped viruses [30]. More recently, IFITM1 has also been implicated in tumorigenesis and there is evidence that it can positively or negatively regulate cell proliferation depending on the tumor cell type [25,27,31,32,40]. In particular, IFITM1 overexpression has been shown to inhibit proliferation in hepatoma cells [29] and its constitutive overexpression has been shown to positively correlate with improved survival in chronic myeloid leukemia patients [25]. In contrast, upregulation of IFITM1 expression has been reported to play a critical role in both the precancerous stage and carcinogenesis in patients with gastric mucosa infected with *Helicobacter pylori* and cervical cancer [33]. In addition, there is evidence that IFITM1 overexpression induces tumor resistance to natural killer (NK) cells in gastric tumor cells and it facilitates migration and invasion of gastric cancer cells [29]. More recently, investigators have reported that overexpression of IFITM1 promotes head and neck tumor invasion in the early stages of disease progression by mediating the expression of molecules downstream, including matrix metalloproteinases 12 and 13 [32].

Our data provide strong evidence that constitutive overexpression of IFITM1 is driven by IFNα through activation of the canonical signaling pathway and that STAT1 and STAT2 play a critical role. In particular, we found that p-STAT1 and p-STAT2 (Figure 3F) were constitutively overexpressed in our resistant cells and that knockdown of IFNα dramatically reduced their expression in the cells. Furthermore, we found that knockdown of STAT1 and STAT2 dramatically reduced IFITM1 expression in the resistant cells. However, we should note that knockdown of IFNα or neutralizing IFNAR1/2 did not completely suppress IFITM1 expression in the resistant cells, which suggests that IFITM1 constitutive overexpression might be regulated by other mechanisms. Indeed, recent studies suggest that IFITM1 expression is regulated by the chromatin remodeling complexes (CRCs) consisting of BRG (Brahma-related gene) and BAF (BRM-associated factor), which work in concert with histone modification enzymes such as cyclic AMP-responsive-element binding protein (CREB)-binding protein and/or p300 (CBP/p300) to bring about the regulation of IFITM1 and other IFNα-target genes [58,59]. The BAF complexes are thought to prime the IFITM1 promoter by disrupting the nucleosome that covers the ISRE which then leads to constitutive expression of IFITM1, as demonstrated in our working model in Figure 9. Future studies will be aimed at determining how IFITM1 and other ISGs are regulated by the BRG/BAF complex in the resistant cells.

## Conclusions

In summary, overexpression of ISGs and their protein products are emerging as important contributors to development of clinical neoplasia and drug resistance in many types of cancers. In our study, we demonstrated that several ISGs including *IFITM1, STAT1* and *PLSCR1* and their encoded proteins are constitutively overexpressed in AI-resistant breast cancer cells and AI-resistant tumors and that their overexpression provides a survival advantage in the resistant cells. Furthermore, we showed that targeting ISGs, especially IFITM1, sensitized AI-resistant breast cancer cells to estrogen-induced cell death and it blocked the ability of these cells to migrate and invade. This finding has important clinical implications for patients with AI-resistant breast cancer because it suggests that altered interferon signaling might play a role in tumor progression and possibly the development of AI resistance. Future studies will need to address how and why interferon response genes are altered during resistance and whether an altered immune response gene profile can predict which patient will benefit from AI therapy and which patient will develop resistance following treatment.

## Additional files

Additional file 1: Figure S1. Overexpression of ISGs in AI-resistant MCF-7:5C cells as compared to parental MCF-7 cells. Total RNA was extracted from each cell line and mRNA expression of the ISGs shown above was determined by real-time PCR using *PUM1* as the internal control. Primer sequences for the ISGs shown above are described in materials and methods. Fold change was calculated using the ΔΔCT method and is displayed as relative to MCF-7 cells (control). Values are means of triplicate measurements ± SD from two independent experiments. Additional file 2: Figure S2. Activation of interferon signaling pathway in parental MCF-7 and AI-resistant MCF-7:5C cells in response to INF-a. **(A)** MCF-7 and **(B)** MCF-7:5C cells were incubated with IFN-α (1000 U/ml) for the indicated time points. The cell extracts were examined by Western blotting using anti-PLSCR1, anti-IFITM1, anti-STAT1, anti-STAT2 and anti-b-actin. The protein levels were quantified using the ImageJ software (downloaded from NIH website) and normalized as the ratio relate to β-actin. **P* <0.05 or ***P* <0.01 versus control. Additional file 3: Figure S3. Activation of IFN signaling pathway in parental MCF-7 and AI-resistant MCF-7:5C cells in response to IFN-α. MCF-7 and MCF-7:5C cells were treated with 100 U/mL IFN-α and harvested at the indicated time points. mRNA expression of IFITM1, PLSCR1 and STAT1was measured using RT-PCR and calculated using the ΔΔCT method relative to PUM1. mRNA expression is given as fold change over control (time zero). Values shown are means of triplicate measurements ± SD from three independent experiments. Additional file 4: Figure S4. IFITM1 knockdown increases cell death in AI-resistant breast cancer cells. (**A**, top panel) MCF-7:5C cells were transfected with control shRNA (shCon), IFITM1 shRNA (shIFITM1) for 24 hours and then treated with 1 nM E2 for an additional 72 hours. Cell extracts were subject to Western blotting for the level of IFITM1 and PARP protein (top panel). Membranes were also stripped and reprobed for β-actin, which was used as a loading control. (**A**, bottom panel) shIFITM1 mRNA level in resistant MCF-7:5C cells was determined by real-time PCR and normalized to *PUM1*. **P* <0.05 versus shcontrol (shCon). **(B)** Cell proliferation was measured in resistant MCF-7:5C cells by MTT assay. All the illustrated data were performed in triplicate and are expressed as mean values of three independent experiments. Standard deviations are shown. **P* <0.05 versus control; #*P* <0.05 versus E2 treatment. **(C)** MCF-7:5C cells were transfected with shCon or shIFITM1 and after 24 hours were exposed to E2 (1 nM) for an additional 72 hours. Cells were then stained with annexin V-FITC and PI for detection of apoptosis as described in Methods. Additional file 5: Figure S5. Role of IFN-α in estradiol-induced cell death in resistant MCF-7:5C cells. Cells were transfected with siIFNα or siCon for 24 hours and then further treated with 1 nM estradiol (E2) for an additional 96 hours. In parallel experiments, we also pre-treated MCF-7:5C cells with 5 μg/mL α-IFNAR antibody (MAB1155) for 4 hours and then treated with 1 nM estradiol for an additional 96 hours. At the 96 hour time point cells were harvested and proliferation was determined by MTT assay (top) and apoptosis was assessed by annexin V/PI staining (bottom). Data shown are expressed as mean values of three independent experiments. Additional file 6: Figure S6. STAT1/STAT2 knockdown reduces IFITM1 expression in MCF-7:5C cells. Cells were transfected with sicontrol (siCon), STAT1 siRNA (siSTAT1), STAT2 siRNA (siSTAT2), or siSTAT1 and siSTAT2 for 48 hours and cells were harvested and analyzed by Western blot to assess STAT1, STAT2 and IFITM1 protein expression. Membranes were stripped and reprobed for β-actin, which was used as a loading control. Blots shown are representative of three separate experiments yielding similar results.

## Abbreviations

AI: aromatase inhibitor
BAF: Brahma-associated factor
BRG: Brahma-related gene
BST2: bone marrow stromal cell antigen 2
CBP/p300: CREB-binding protein 300
CRCs: chromatin remodeling complexes
CREB: cyclic AMP-responsive-element binding protein
DAB: 3,3′-diaminobenzidine
(D)MEM: (Dulbecco’s) modified Eagle’s medium
DMSO: dimethyl sulfoxide
E_2_: 17β-estradiol
ECL: enhanced chemiluminescence
ELISA: enzyme-linked immunosorbent assay
ER: estrogen receptor
ERα: estrogen receptor alpha
ER+: estrogen receptor-positive
ERE: estrogen response element
FBS: fetal bovine serum
HRP: horseradish peroxidase
ICC: immunocytochemistry
IF: immunofluorescence
IFI27: interferon-inducible protein 27
IFIT1: Interferon-induced protein with tetratricopeptide repeats 1
IFITM1: interferon induced transmembrane protein1
IFNs: interferons
IFNR1/2: interferon receptor type 1 and 2
IgG: immunoglobulin G
IHC: immunohistochemistry
IR F-7: IFN regulatory factor 7
IRF9: IFN regulatory factor 9
ISGF3: IFN-stimulated gene factor 3
ISGs: interferon stimulated genes
ISREs: interferon stimulated response elements
JAK: Janus kinase 1 and 2
MTT: 3-(4,5-dimethylthiazol-2-Yl)-2,5-diphenyltetrazolium bromide
NK: natural killer
OAS 1,2,3: 2′-5′-oligoadenylate synthetase 1,2,3, PARP, poly ADP ribose polymerase
PBS: phosphate-buffered saline
PCR: polymerase chain reaction
PI: propidium iodide
PLSCR1: phospholipid scramblase 1
SDS-PAGE: sodium dodecyl sulfate-polyacrylamide gel electrophoresis
SI: staining index
SOCS1: suppressor of cytokine signaling 1
STAT1,2: signal transducer and activator of transcription 1,2
TTP: time to disease progression
U-ISGF3: unphosphorylated IFN-stimulated gene factor 3
4OHT: 4-hydoxytamoxifen
