# Supplementary material for: Targeting interferon response genes sensitizes aromatase inhibitor resistant breast cancer cells to estrogen-induced cell death
Source: Breast Cancer Res. 2015 Jan 15;17(1):6. doi: 10.1186/s13058-014-0506-7 (PMC4336497; doi:10.1186/s13058-014-0506-7)
Supplement: Additional file 1: Figure S1. — Overexpression of ISGs in AI-resistant MCF-7:5C cells as compared to parental MCF-7 cells. Total RNA was extracted from each cell line and mRNA expression of the ISGs shown above was determined by real-time PCR using PUM1 as the internal control. Primer sequences for the ISGs shown above are described in materials and methods. Fold change was calculated using the ΔΔCT method and is displayed as relative to MCF-7 cells (control). Values are means of triplicate measurements ± SD from two independent experiments. [file 13058_2014_506_MOESM1_ESM.ppt]

## Slide 1
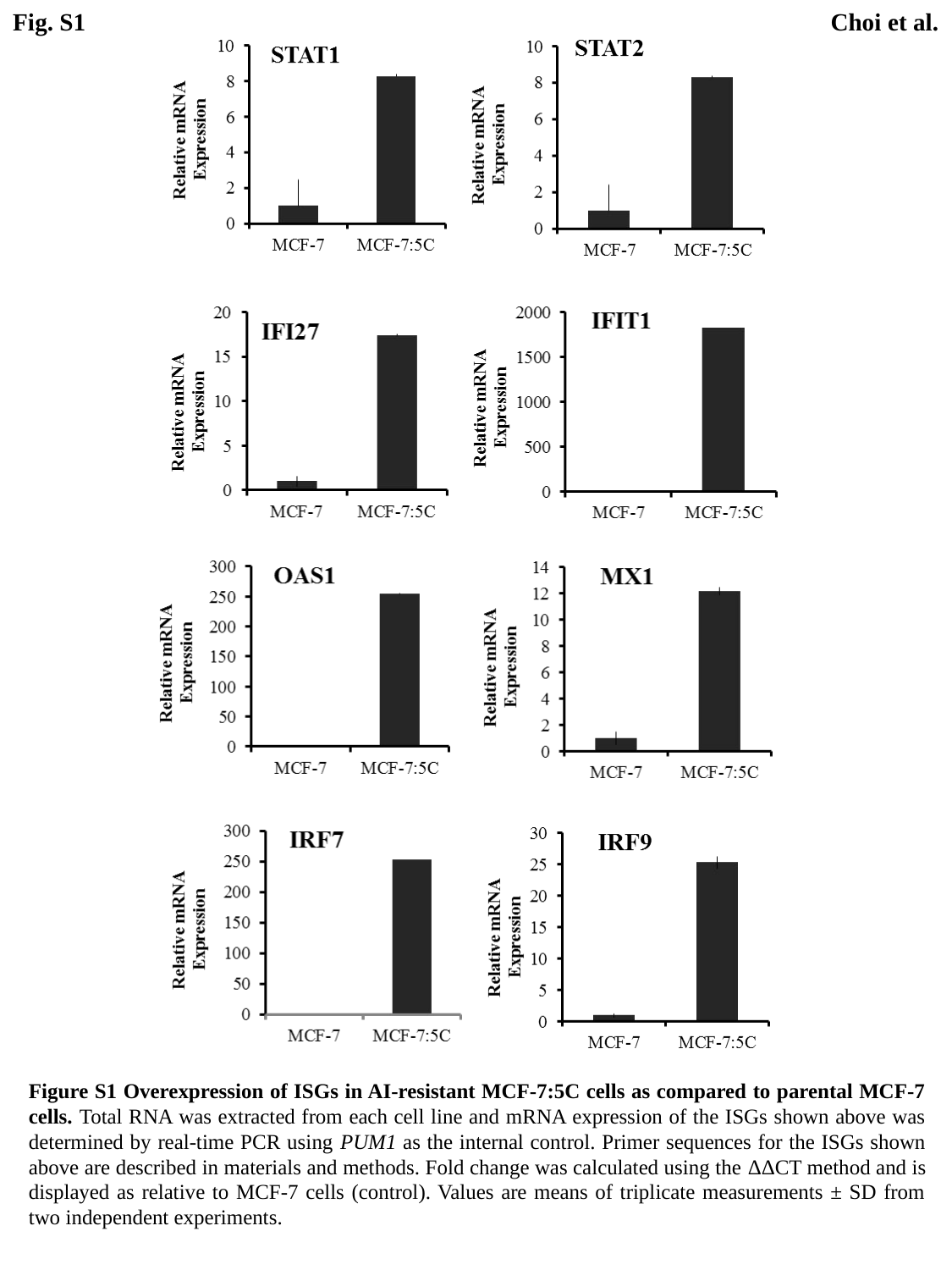

Fig. S1
Choi et al.
Figure S1 Overexpression of ISGs in AI-resistant MCF-7:5C cells as compared to parental MCF-7 cells. Total RNA was extracted from each cell line and mRNA expression of the ISGs shown above was determined by real-time PCR using PUM1 as the internal control. Primer sequences for the ISGs shown above are described in materials and methods. Fold change was calculated using the ΔΔCT method and is displayed as relative to MCF-7 cells (control). Values are means of triplicate measurements ± SD from two independent experiments.
