# Supplementary material for: Targeting interferon response genes sensitizes aromatase inhibitor resistant breast cancer cells to estrogen-induced cell death
Source: Breast Cancer Res. 2015 Jan 15;17(1):6. doi: 10.1186/s13058-014-0506-7 (PMC4336497; doi:10.1186/s13058-014-0506-7)
Supplement: Additional file 2: Figure S2. — Activation of interferon signaling pathway in parental MCF-7 and AI-resistant MCF-7:5C cells in response to INF-a. (A) MCF-7 and (B) MCF-7:5C cells were incubated with IFN-α (1000 U/ml) for the indicated time points. The cell extracts were examined by Western blotting using anti-PLSCR1, anti-IFITM1, anti-STAT1, anti-STAT2 and anti-b-actin. The protein levels were quantified using the ImageJ software (downloaded from NIH website) and normalized as the ratio relate to β-actin. *P <0.05 or **P <0.01 versus control. [file 13058_2014_506_MOESM2_ESM.ppt]

## Slide 1
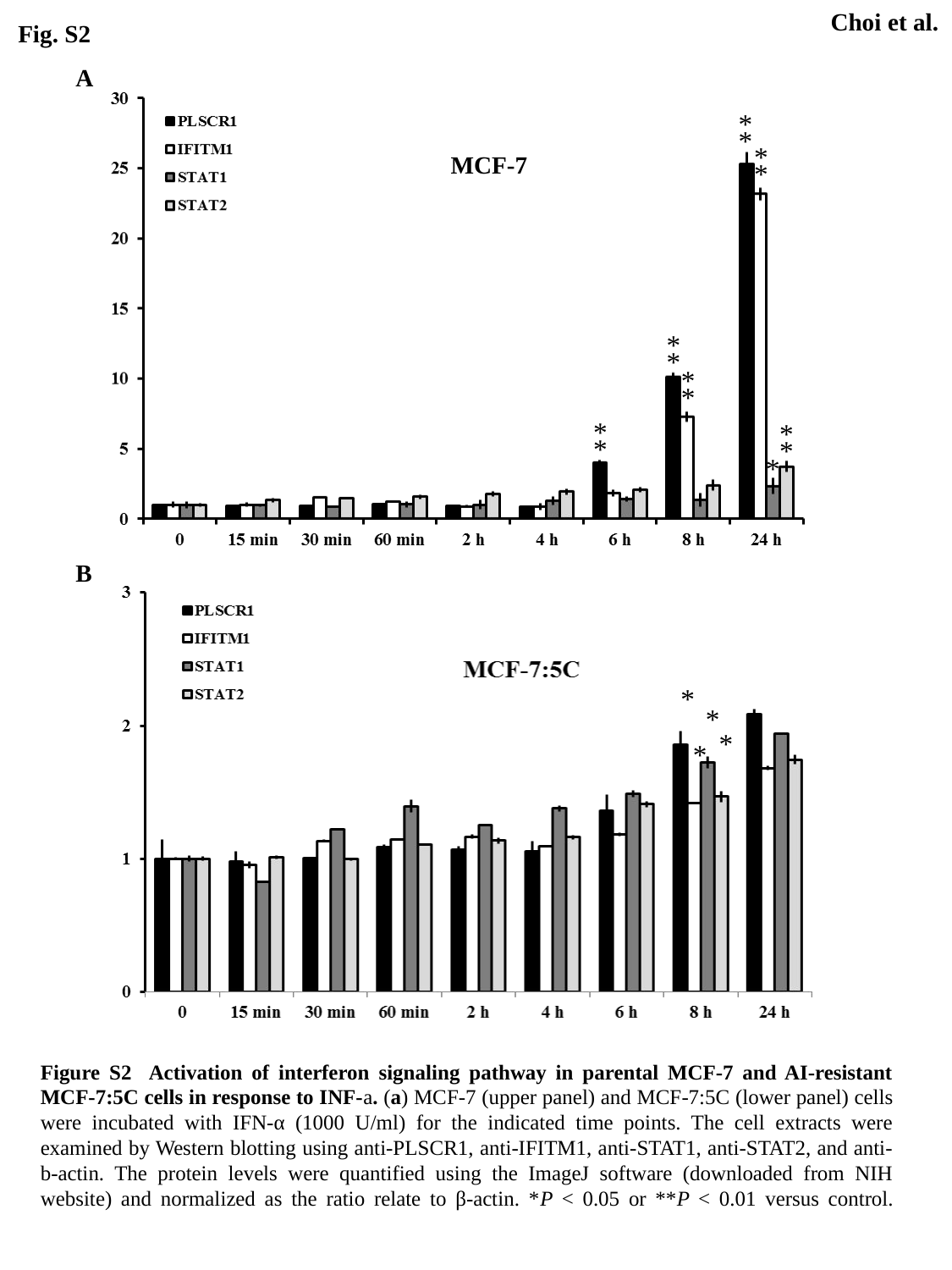

Choi et al.
Fig. S2
A
*
*
*
*
MCF-7
*
*
*
*
*
*
*
*
*
B
*
*
*
*
Figure S2 Activation of interferon signaling pathway in parental MCF-7 and AI-resistant MCF-7:5C cells in response to INF-a. (a) MCF-7 (upper panel) and MCF-7:5C (lower panel) cells were incubated with IFN-α (1000 U/ml) for the indicated time points. The cell extracts were examined by Western blotting using anti-PLSCR1, anti-IFITM1, anti-STAT1, anti-STAT2, and anti-b-actin. The protein levels were quantified using the ImageJ software (downloaded from NIH website) and normalized as the ratio relate to β-actin. *P < 0.05 or **P < 0.01 versus control.
