# Supplementary material for: Targeting interferon response genes sensitizes aromatase inhibitor resistant breast cancer cells to estrogen-induced cell death
Source: Breast Cancer Res. 2015 Jan 15;17(1):6. doi: 10.1186/s13058-014-0506-7 (PMC4336497; doi:10.1186/s13058-014-0506-7)
Supplement: Additional file 3: Figure S3. — Activation of IFN signaling pathway in parental MCF-7 and AI-resistant MCF-7:5C cells in response to IFN-α. MCF-7 and MCF-7:5C cells were treated with 100 U/mL IFN-α and harvested at the indicated time points. mRNA expression of IFITM1, PLSCR1 and STAT1was measured using RT-PCR and calculated using the ΔΔCT method relative to PUM1. mRNA expression is given as fold change over control (time zero). Values shown are means of triplicate measurements ± SD from three independent experiments. [file 13058_2014_506_MOESM3_ESM.ppt]

## Slide 1
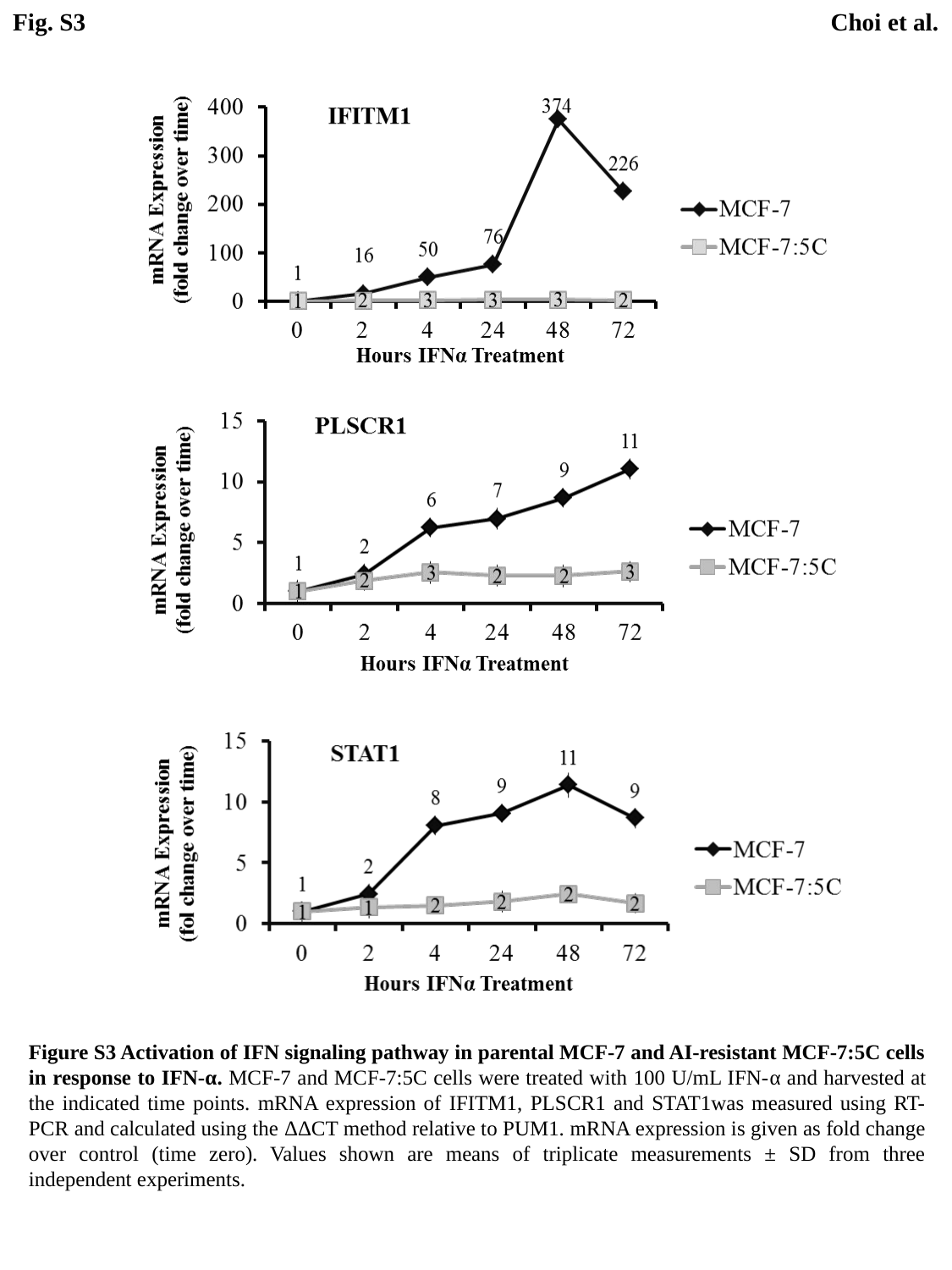

Fig. S3
Choi et al.
Figure S3 Activation of IFN signaling pathway in parental MCF-7 and AI-resistant MCF-7:5C cells in response to IFN-α. MCF-7 and MCF-7:5C cells were treated with 100 U/mL IFN-α and harvested at the indicated time points. mRNA expression of IFITM1, PLSCR1 and STAT1was measured using RT-PCR and calculated using the ΔΔCT method relative to PUM1. mRNA expression is given as fold change over control (time zero). Values shown are means of triplicate measurements ± SD from three independent experiments.
