# Supplementary material for: Targeting interferon response genes sensitizes aromatase inhibitor resistant breast cancer cells to estrogen-induced cell death
Source: Breast Cancer Res. 2015 Jan 15;17(1):6. doi: 10.1186/s13058-014-0506-7 (PMC4336497; doi:10.1186/s13058-014-0506-7)
Supplement: Additional file 4: Figure S4. — IFITM1 knockdown increases cell death in AI-resistant breast cancer cells. (A, top panel) MCF-7:5C cells were transfected with control shRNA (shCon), IFITM1 shRNA (shIFITM1) for 24 hours and then treated with 1 nM E2 for an additional 72 hours. Cell extracts were subject to Western blotting for the level of IFITM1 and PARP protein (top panel). Membranes were also stripped and reprobed for β-actin, which was used as a loading control. (A, bottom panel) shIFITM1 mRNA level in resistant MCF-7:5C cells was determined by real-time PCR and normalized to PUM1. *P <0.05 versus shcontrol (shCon). (B) Cell proliferation was measured in resistant MCF-7:5C cells by MTT assay. All the illustrated data were performed in triplicate and are expressed as mean values of three independent experiments. Standard deviations are shown. *P <0.05 versus control; #P <0.05 versus E2 treatment. (C) MCF-7:5C cells were transfected with shCon or shIFITM1 and after 24 hours were exposed to E2 (1 nM) for an additional 72 hours. Cells were then stained with annexin V-FITC and PI for detection of apoptosis as described in Methods. [file 13058_2014_506_MOESM4_ESM.ppt]

## Slide 1
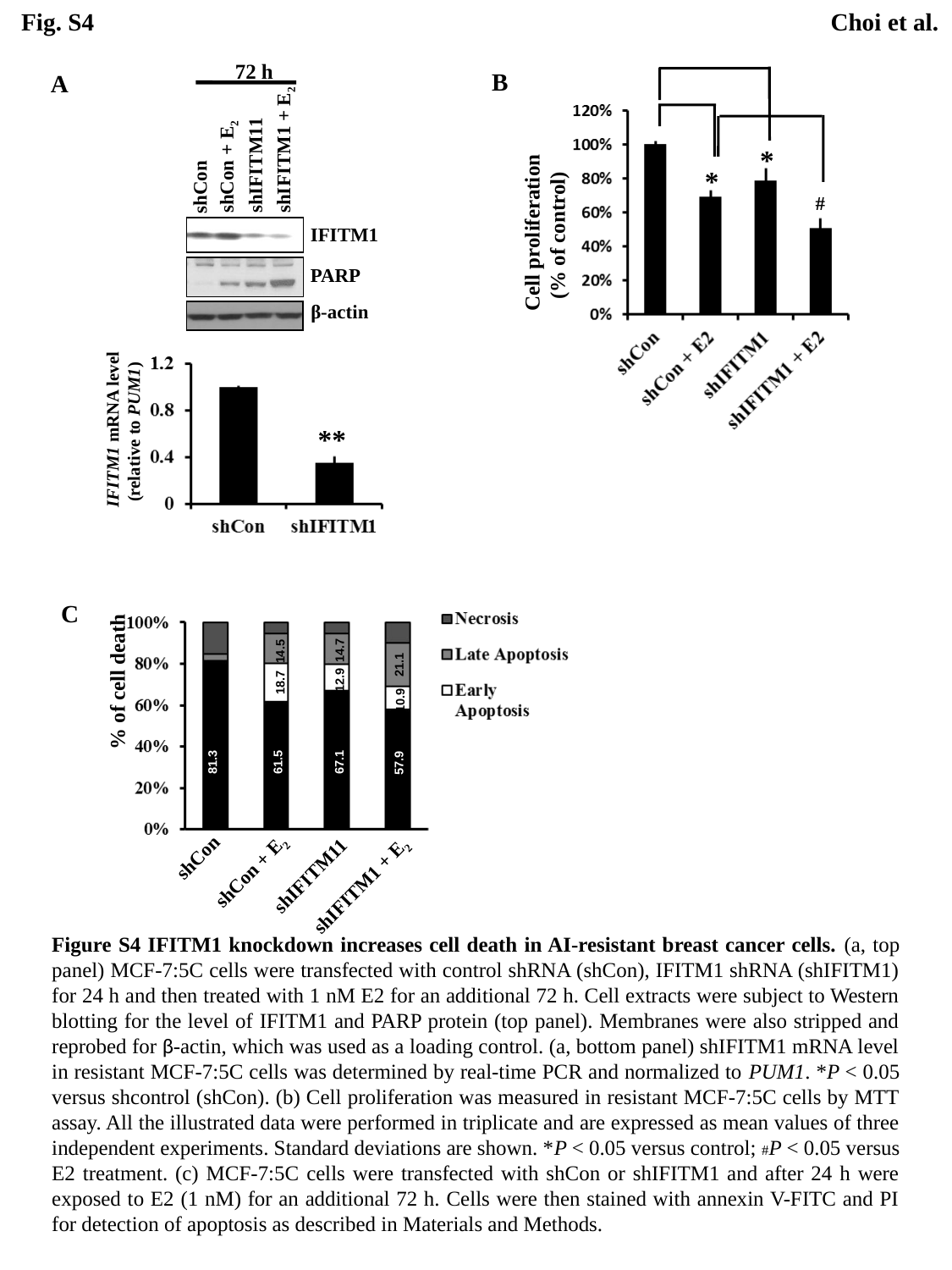

Fig. S4
Choi et al.
72 h
shIFITM1 + E2
shCon + E2
shIFITM11
shCon
IFITM1
PARP
β-actin
B
*
#
*
Cell proliferation
(% of control)
A
IFITM1 mRNA level
(relative to PUM1)
**
C
14.7
14.5
21.1
12.9
18.7
10.9
81.3
61.5
67.1
57.9
shCon
shCon + E2
shIFITM11
shIFITM1 + E2
% of cell death
Figure S4 IFITM1 knockdown increases cell death in AI-resistant breast cancer cells. (a, top panel) MCF-7:5C cells were transfected with control shRNA (shCon), IFITM1 shRNA (shIFITM1) for 24 h and then treated with 1 nM E2 for an additional 72 h. Cell extracts were subject to Western blotting for the level of IFITM1 and PARP protein (top panel). Membranes were also stripped and reprobed for β-actin, which was used as a loading control. (a, bottom panel) shIFITM1 mRNA level in resistant MCF-7:5C cells was determined by real-time PCR and normalized to PUM1. *P < 0.05 versus shcontrol (shCon). (b) Cell proliferation was measured in resistant MCF-7:5C cells by MTT assay. All the illustrated data were performed in triplicate and are expressed as mean values of three independent experiments. Standard deviations are shown. *P < 0.05 versus control; #P < 0.05 versus E2 treatment. (c) MCF-7:5C cells were transfected with shCon or shIFITM1 and after 24 h were exposed to E2 (1 nM) for an additional 72 h. Cells were then stained with annexin V-FITC and PI for detection of apoptosis as described in Materials and Methods.
