# Supplementary material for: Targeting interferon response genes sensitizes aromatase inhibitor resistant breast cancer cells to estrogen-induced cell death
Source: Breast Cancer Res. 2015 Jan 15;17(1):6. doi: 10.1186/s13058-014-0506-7 (PMC4336497; doi:10.1186/s13058-014-0506-7)
Supplement: Additional file 5: Figure S5. — Role of IFN-α in estradiol-induced cell death in resistant MCF-7:5C cells. Cells were transfected with siIFNα or siCon for 24 hours and then further treated with 1 nM estradiol (E2) for an additional 96 hours. In parallel experiments, we also pre-treated MCF-7:5C cells with 5 μg/mL α-IFNAR antibody (MAB1155) for 4 hours and then treated with 1 nM estradiol for an additional 96 hours. At the 96 hour time point cells were harvested and proliferation was determined by MTT assay (top) and apoptosis was assessed by annexin V/PI staining (bottom). Data shown are expressed as mean values of three independent experiments. [file 13058_2014_506_MOESM5_ESM.ppt]

## Slide 1
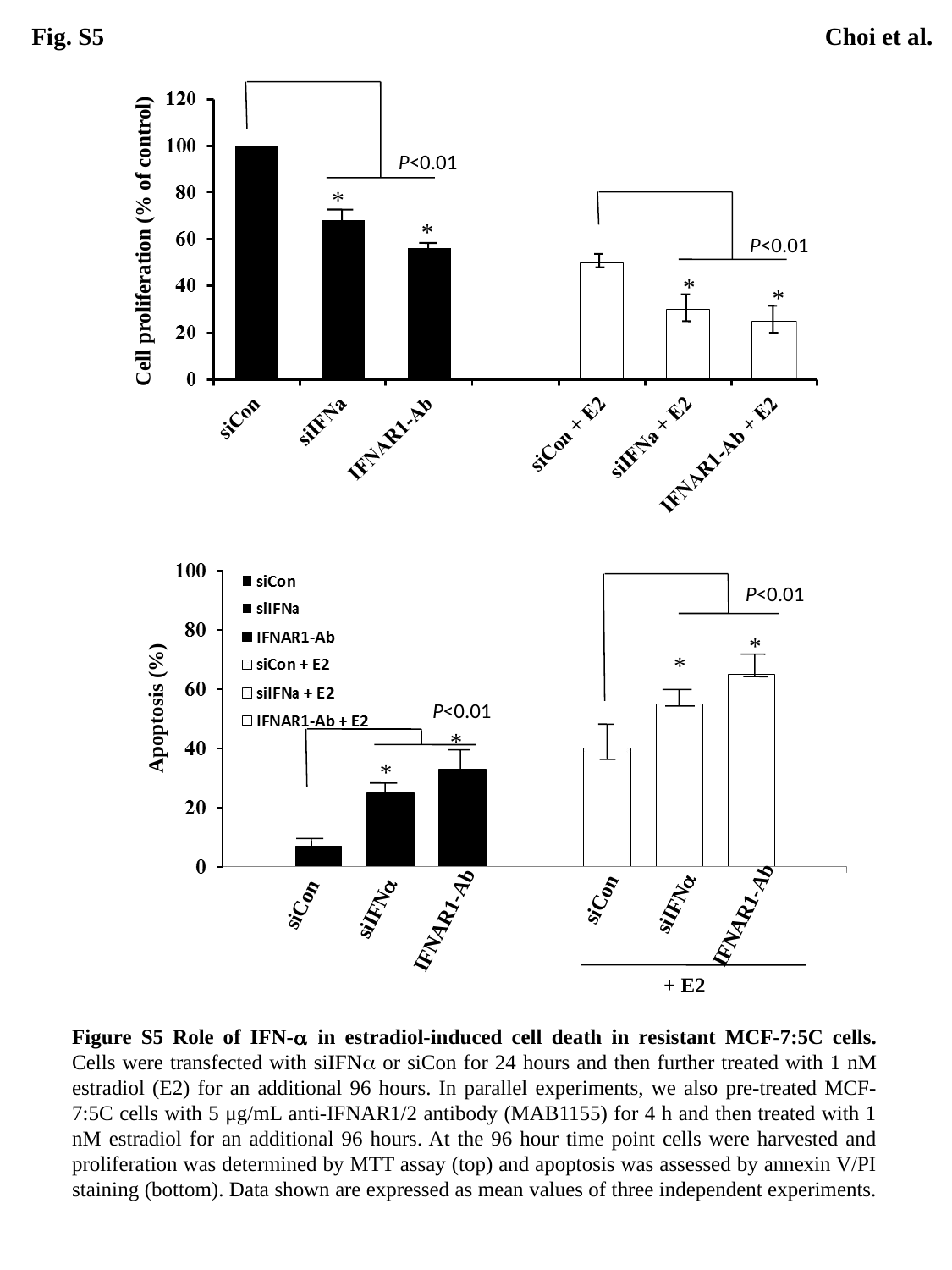

Fig. S5
Choi et al.
P<0.01
*
*
Cell proliferation (% of control)
P<0.01
*
*
siCon
siIFNα
siCon
siIFNα
IFNAR1-Ab
IFNAR1-Ab
+ E2
P<0.01
*
*
Apoptosis (%)
P<0.01
*
*
Figure S5 Role of IFN-α in estradiol-induced cell death in resistant MCF-7:5C cells. Cells were transfected with siIFNα or siCon for 24 hours and then further treated with 1 nM estradiol (E2) for an additional 96 hours. In parallel experiments, we also pre-treated MCF-7:5C cells with 5 μg/mL anti-IFNAR1/2 antibody (MAB1155) for 4 h and then treated with 1 nM estradiol for an additional 96 hours. At the 96 hour time point cells were harvested and proliferation was determined by MTT assay (top) and apoptosis was assessed by annexin V/PI staining (bottom). Data shown are expressed as mean values of three independent experiments.
