# Supplementary material for: Targeting interferon response genes sensitizes aromatase inhibitor resistant breast cancer cells to estrogen-induced cell death
Source: Breast Cancer Res. 2015 Jan 15;17(1):6. doi: 10.1186/s13058-014-0506-7 (PMC4336497; doi:10.1186/s13058-014-0506-7)
Supplement: Additional file 6: Figure S6. — STAT1/STAT2 knockdown reduces IFITM1 expression in MCF-7:5C cells. Cells were transfected with sicontrol (siCon), STAT1 siRNA (siSTAT1), STAT2 siRNA (siSTAT2), or siSTAT1 and siSTAT2 for 48 hours and cells were harvested and analyzed by Western blot to assess STAT1, STAT2 and IFITM1 protein expression. Membranes were stripped and reprobed for β-actin, which was used as a loading control. Blots shown are representative of three separate experiments yielding similar results. [file 13058_2014_506_MOESM6_ESM.ppt]

## Slide 1
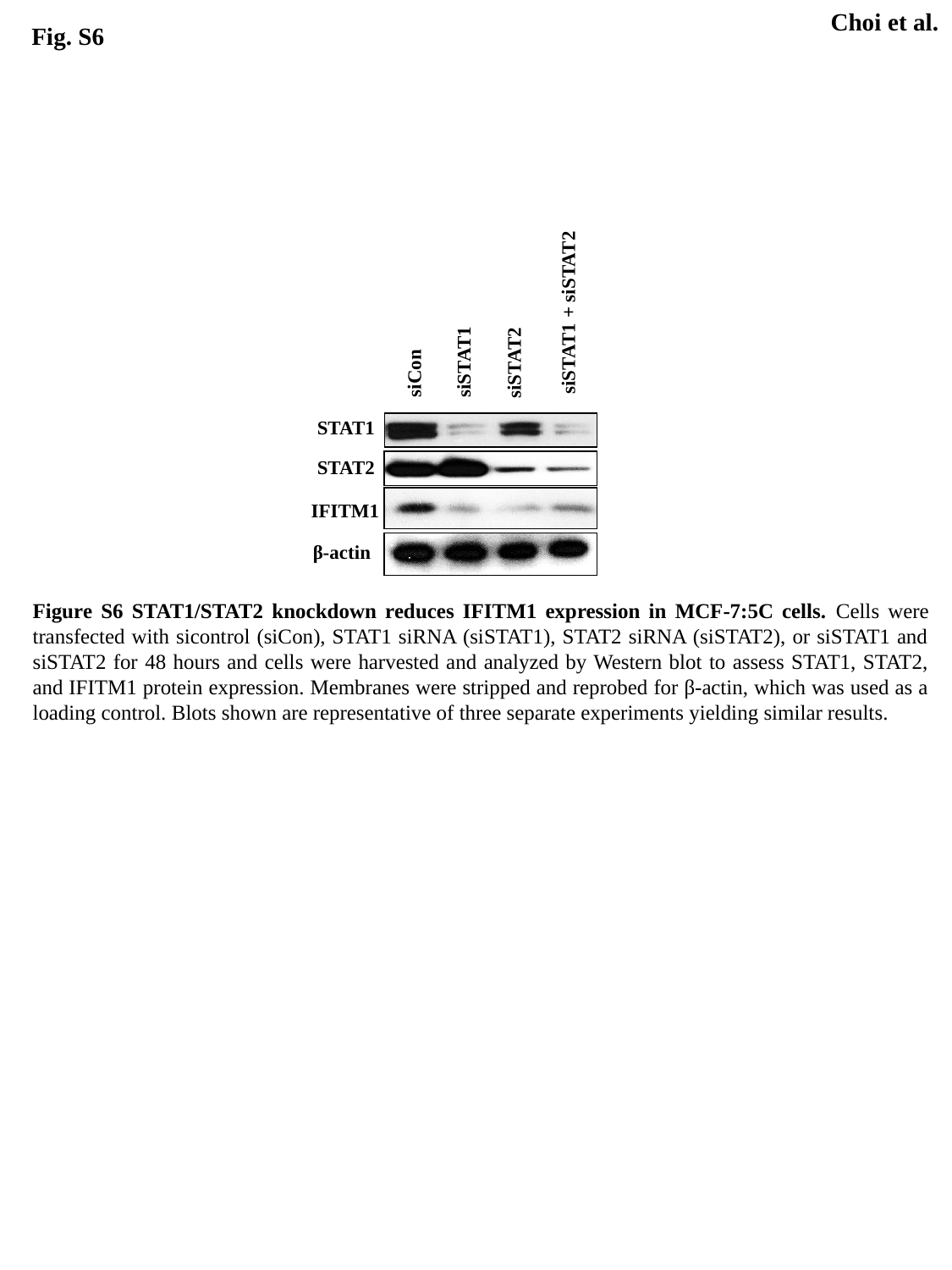

Choi et al.
Fig. S6
siSTAT1 + siSTAT2
siSTAT1
siSTAT2
siCon
STAT1
STAT2
IFITM1
β-actin
Figure S6 STAT1/STAT2 knockdown reduces IFITM1 expression in MCF-7:5C cells. Cells were transfected with sicontrol (siCon), STAT1 siRNA (siSTAT1), STAT2 siRNA (siSTAT2), or siSTAT1 and siSTAT2 for 48 hours and cells were harvested and analyzed by Western blot to assess STAT1, STAT2, and IFITM1 protein expression. Membranes were stripped and reprobed for β-actin, which was used as a loading control. Blots shown are representative of three separate experiments yielding similar results.
